# Overexpression of miR-30b in the Developing Mouse Mammary Gland Causes a Lactation Defect and Delays Involution

**DOI:** 10.1371/journal.pone.0045727

**Published:** 2012-09-24

**Authors:** Sandrine Le Guillou, Nezha Sdassi, Johann Laubier, Bruno Passet, Marthe Vilotte, Johan Castille, Denis Laloë, Jacqueline Polyte, Stéphan Bouet, Florence Jaffrézic, Edmond-Paul Cribiu, Jean-Luc Vilotte, Fabienne Le Provost

**Affiliations:** INRA, UMR1313 Génétique Animale et Biologie Intégrative, Jouy-en-Josas, France; Ecole Normale Supérieure de Lyon, France

## Abstract

**Background:**

MicroRNA (miRNA) are negative regulators of gene expression, capable of exerting pronounced influences upon the translation and stability of mRNA. They are potential regulators of normal mammary gland development and of the maintenance of mammary epithelial progenitor cells. This study was undertaken to determine the role of miR-30b on the establishment of a functional mouse mammary gland. miR-30b is a member of the miR-30 family, composed of 6 miRNA that are highly conserved in vertebrates. It has been suggested to play a role in the differentiation of several cell types.

**Methodology/Principal Findings:**

The expression of miR-30b was found to be regulated during mammary gland development. Transgenic mice overexpressing miR-30b in mammary epithelial cells were used to investigate its role. During lactation, mammary histological analysis of the transgenic mice showed a reduction in the size of alveolar lumen, a defect of the lipid droplets and a growth defect of pups fed by transgenic females. Moreover some mammary epithelial differentiated structures persisted during involution, suggesting a delay in the process. The genes whose expression was affected by the overexpression of miR-30b were characterized by microarray analysis.

**Conclusion/Significance:**

Our data suggests that miR-30b is important for the biology of the mammary gland and demonstrates that the deregulation of only one miRNA could affect lactation and involution.

## Introduction

MicroRNA (miRNA), endogenous small RNA (18–22 nt), are negative regulators of gene expression, capable of exerting pronounced influences upon the translation and stability of mRNA [1], [2]. miRNA-mediated gene regulation is crucial for many biological processes such as cellular growth, cell differentiation, death or polarity [3]. Approximately 1,000 human miRNA have been identified to date [4], but this number might have been over-estimated [5]. Each miRNA might target several hundreds of distinct mRNA molecules [6], suggesting that the majority of mRNA are subject to regulation by miRNA. Despite great advances, the miRNA field remains largely uncharted with regards to the physiological function of these small molecules.

The miR-30 family is highly conserved in Vertebrates, it is composed by 6 miRNA (miR-30a, -30b, -30c-1, -30c-2, -30d and -30e) and it is organized in 3 clusters of two miRNA localized on 3 different chromosomes. A role of this miRNA family has been suggested in the differentiation of adipocytes [7], of B-cells [8], of osteoblasts [9], in the control of the pluripotency of embryonic stem cells [10] and in the control of the epithelial-to-mesenchyme transition (EMT) [11], [12], [13]. The miR-30 family is also involved in the control of structural changes in the extracellular matrix of the myocardium [14], in cellular senescence [15] and in the regulation of the apoptosis [16]. In cancer processes, this miRNA family has been suggested to be important in the development of medulloblastoma [17] and melanoma metastasis [18]. miR-30b, in particular, is considered to be a tumor-suppressor miRNA [19]. It is regulated by estrogen in breast cancer cells [20], associated with mammary tumorigenesis and metastasis [21], and it is one of the 13 miRNA whose expression of which differs between inflammatory breast cancer (IBC) and non IBC [22]. Its expression has been detected in several human mammary cell types, as basal and luminal epithelial cells and fibroblasts [23], [24].

The mammary gland undergoes an important development of its epithelial tissues after birth under the control of the female reproductive hormones. At birth, a rudimentary ductal system is present which extends to the fat pad tissue until it reaches the periphery of the gland at puberty. Alveolar structures develop at the ends of the side-branches and differentiate into lobuloalveolar structures during gestation to become sites of milk production during lactation. After weaning, the gland involutes which causes massive cell death, alveoli collapse and remodeling of the epithelial compartment to restore a simple ductal structure like that of the virgin stage (reviewed in [25], [26]).

Although deregulation of miRNA in the setting of breast cancer has been well documented, relatively little is known about the role of miRNA during normal mammary gland development. Recently, Ucar and colleagues [27] have shown that miR-212 and miR-132 are indispensable during the development of the mammary gland. A link between miRNA and mammary epithelial progenitor cells was evidenced using the mouse mammary epithelial cell line COMMA-Dβ [28], [29]. Regulation of both EMT and EMT-associated stem cell properties via tumor suppressor p53-mediated transcriptional activation of miR-200c was also demonstrated [30].

Here, we used transgenic mice overexpressing miR-30b to investigate its role in the development and differentiation of mammary epithelial cells *in vivo* and we show that miR-30b deregulation leads to an impairment of mammary gland structure and function during lactation and involution.

## Materials and Methods

### Animals and tissue collection

All animal manipulations were performed according to the French “Commission de Génie Génétique” recommendations. Animals were bred in a controlled environment and treated within published guidelines of human animal care. The day of the vaginal plug appearance was counted as day-0 of gestation. For mammary gland lactation, the day of delivery was considered as day-0. For involution, dams were allowed to lactate for 5 days or more, and the pups were removed (day-0) to induce involution. When glands (number 4) were collected for RNA analysis, the lymph nodes were removed before homogenization. For the pup growth curve analysis, the number of pups per litter was reduced to eight after parturition.

### Transgenesis

The mouse precursor of miR-30b (pre-miR-30b) was under the control of MMTV-LTR. The oligonucleotides 5′-TCCCCCGGGGGCTAAGCCAAGTTTCAGTTCATGTAAACATCCTACACTCAGCTGTCATACATGCGTTGGC-3′ and 5′-CCGCTCGAGCGGATACTCCAAGACAGCTGACGTAAACATCCACACCCAGCCAACGCATGTATGACAGCTGAGTG-3′ were annealed, filled with the Klenow fragment enzyme and digested with *Sma*I and *Xho*I. The resulting fragment was inserted into the corresponding sites of the pMSG plasmid (Amersham Pharmacia Biotech). The *Hind*III insert was released from this vector, purified, diluted in TE (10-0.1) pH 7.4 at a final concentration of 2 ng/µl and microinjected into pronuclei of FVB/N mouse eggs.

Transgenic mice were identified by PCR of genomic DNA extracted from tail biopsies. Transgene-specific amplification was performed using a set of oligonucleotides: pmir30b-sens: 5′-TCCCCCGGGGGCTAAGCCAAGTTTCAGTTCATGTAAACATCCTACACTCAGCTGTCATACATGCGTTGGC-3′ and pMSG/1: 5′-GTCACACCACAGAAGTAAGG-3′, and the GoTaq Flexi DNA polymerase kit (Promega) according to the following PCR program: 94°C for 5 min followed by 30 cycles of 3 temperature steps (94°C for 30 s, 58°C for 30 s and 72°C for 45 s).

### RNA isolation

Total RNA was isolated from mouse tissue biopsies with the RNA NOW kit (Ozyme), with an overnight precipitation, so as to guarantee a maximum yield of miRNA. The RNA quantity and quality were assessed using an Agilent BioAnalyzer.

### Microarray analysis

100 ng of total RNA was labeled following the manufacturer's protocols and hybridized to Affymetrix® Mouse Gene 1.1 ST Array, representing 28,000 well-annotated genes with more than 770,000 distinct probe sets. It was carried out at the Affymetrix Platform of Institut Curie, Paris. Experiments were performed with RNA extracted from the mammary glands of 12 individual lactating (day-12) mice (4 wild-type, 4 Tg12 and 4 Tg33 lines) and of 11 individual mice at involution day-6 stage (4 wild-type, 4 Tg12 and 3 Tg33 lines).

### Quantitative PCR

Quantifications of mRNA were performed by reverse transcription (RT) of 5 µg of total RNA using the Superscript First Strand Synthesis System II (Invitrogen) following the manufacturer's instructions. Quantitative PCR (qPCR) was performed on RT products using the Mastercycler ep Realplex (Eppendorf). Reaction conditions consisted of 15 min at 95°C (1 cycle), 15 s at 95°C and 60 s at 60°C (45 cycles) with primers (10 µM) using ABsolute QPCR SYBR Green (Thermo Scientific). Primer sequences are presented in Table S1. Each sample was analyzed in triplicate for 3 transgenic and 3 wild-type animals. After normalization for the housekeeping gene: *Gapdh* for lactation day-12 stage and *Tbrg4* [31] for involution day-6 samples, expression levels were compared between transgenic and control samples using the Delta-Delta Ct method (2^−ΔΔCt^) [32].

miRNA were quantified by RT-qPCR using TaqMan MicroRNA Expression Assays (Applied Biosystem). Briefly, 5 ng of total RNA (pooled from 3 animals) were reverse transcribed under the following conditions: 16°C for 30 min, 42°C for 30 min, 85°C for 5 min. PCR reactions were as follows: 95°C for 10 min followed by 40 cycles of 95°C for 15 s and 60°C for 1 min using the Mastercycler ep Realplex (Eppendorf). *RNU6B* was used for normalization (Applied Biosystems). The relative abundance was multiplied by 1,000.

### Histological, immunohistochemistry, and TUNEL assay

For histological analysis, dissected mammary glands were fixed in RCl2 (Alphelys) and embedded in paraffin. Paraffin sections (5 µm) were used for hematoxylin-eosin-saffran (HES) staining.

For immunohistochemical detection of Ki67, heat-induced retrieval was performed by microwaving sections in 10 mM sodium citrate, pH 6.0 for 10 min. After blocking in 0.05% fetal bovine serum (Lonza), sections were incubated overnight at 4°C with primary antibodies (Rabbit monoclonal anti-Ki67, 1∶200; Neo Markers; Cy3-conjuguate mouse monoclonal anti α-smooth muscle actin (α-SMA), 1∶800, Sigma-Aldrich), followed by incubation for 1 h at room temperature with secondary antibody (Goat Alexafluor 488 anti-rabbit, 1∶2000, Invitrogen), then counterstained with Vectashield-DAPI medium (Vectorlabs).

TUNEL staining was carried out on histological sections using the *in situ* Cell Death Detection kit (Roche Applied Sciences).

The immunofluorescence was viewed under a Leica microscope and the quantification of labelled cells was performed using the ImageJ software (RSB) using 5 pictures per sample.

### Lipid droplet staining

Pieces of mammary gland biopsies (2–3 mm) were fixed in 4% paraformaldehyde for 10 min at 4°C, then washed twice with cold 1× PBS for 15 min. Specimens were transferred into 40% sucrose for 6 hours at 4°C, then frozen in Cryomount (Histolab) using liquid nitrogen vapors. Nile Red, a selective fluorescent stain for intracellular lipids, was dissolved in 0.5% isopropanol, and a 1∶1000 dilution in 1× PBS was used to stain frozen sections (7 µm) of mammary gland at room temperature. The sections were washed with PBS containing 0.1% Tween for 5 min and counterstained with Vectashield-DAPI medium. The staining was analysed by fluorescent light microscopy. The quantification and the area of labelled lipid droplets and nuclei were performed using the ImageJ software using 10 pictures per sample.

### Statistical analysis

Differences in expression data obtained by qPCR between control and transgenic lines were compared using a one-way analysis of variance (ANOVA). Mean and Standard Error (S.E.) were calculated for each group. Microsoft Excel (Microsoft) software was used for these analyses and a p-value of 0.05 was considered to be statistically significant.

The microarray data were preprocessed using RMA in the default configuration for background adjustment and normalization. Analyses were done in the BioConductor version 2.10 [33] and R version 2.15.0 environments [34]. To identify genes that were differentially expressed, we applied the empirical Bayes, moderated *t*-statistics implemented in the BioConductor package LIMMA (version 3.12.0) [35]. P-values were adjusted for multiple testing using the Benjamini and Hochberg method [36]. A first differential analysis between the two transgenic lines showed that they significantly differed for 10 genes at an adjusted p-value of 0.05 (*Pappa2, Aqp9, Spcs3, Zdhhc21, Capza1, Unc5b, Olfr1338, Bcl2l11* and two non annotated ones) and only one at an adjusted p-value of 0.01 (*Pappa2*) at lactation day-12, and just one gene at an adjusted p-values of 0.05 and 0.01 at involution day-6 (*Pappa2*). On this basis, for greater depth and robustness, we chose to cumulate the data from the two transgenic lines for the differential analysis against the wild-type data.

## Results and Discussion

### Generation of miR-30b transgenic mice

**Figure 1:**
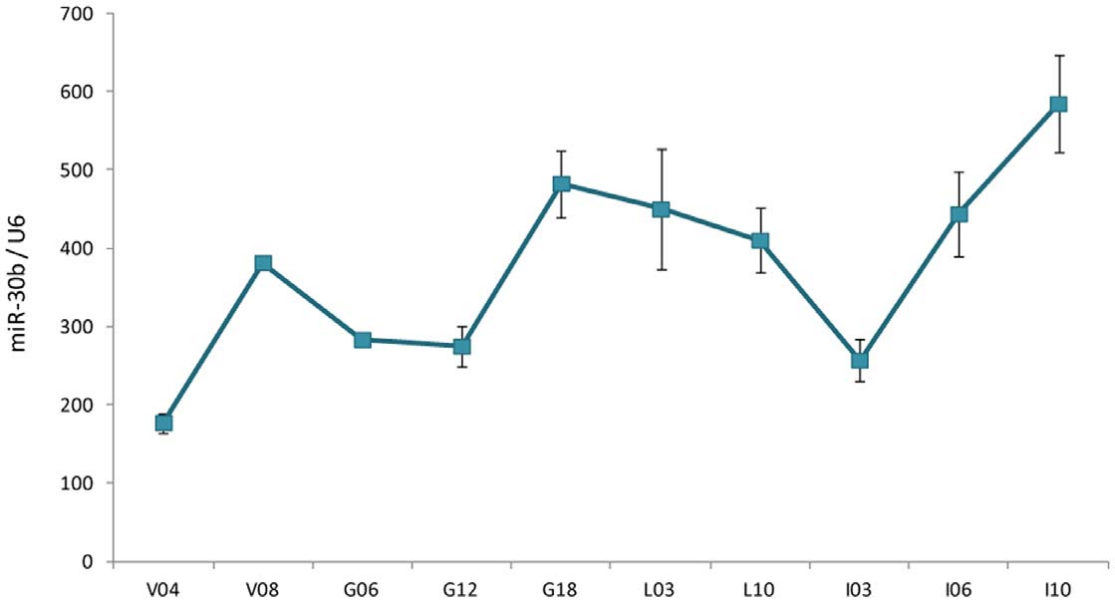
miR-30b expression changes during mouse mammary gland development. Relative expression of miR-30b was determined by RT-qPCR at different physiological stages. miR-30b expression was normalized to *U6* expression. Virgin 4 weeks (V04), 8 weeks (V08), Gestation (G), Lactation (L) and Involution (I) time points (in days). Values are means ± S.E. (n = 3 technical repetitions on pool of 3 mice).

The levels of endogenous miR-30b during mammary gland development and differentiation were assessed by RT-qPCR. miR-30b expression was detected at all stages (Figure 1). Its expression significantly (p<0.05) increased between puberty (4 weeks) and mature virgin (8 weeks) stages; and between mid and end of gestation. During lactation, the level remained stable and it significantly (p<0.05) decreased in early involution. At last, its expression significantly (p<0.05) increased during the late stages of involution. Its overall level of expression and its variability between developmental stages are within the range of those detected for other miRNA (see Figure S1), suggesting physiological significance. This profile is consistent with the results of others obtained by bead-based flow-cytometric profiling [37] and suggests a developmental regulation of miR-30b in the mammary gland.

**Figure 2:**
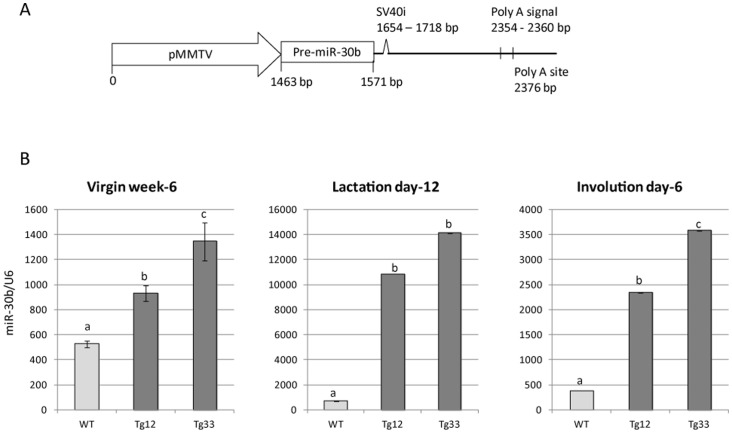
miR-30b expression level is strongly higher during lactation than virgin stage or during involution in mammary gland of transgenic mice. A/ Schematic representation of the transgene. pMMTV = mouse mammary tumor virus promoter, Pre-miR-30b = precursor of miR-30b, SV40i = SV40 small T antigen intron. The figure is not to scale. B/ Relative expression of miR-30b was determined by RT-qPCR at 3 different physiological stages (virgin week-6, lactation day-12 and involution day-6) in the two transgenic lines (Tg12 and Tg33). miR-30b expression was normalized to *U6* expression. Bars and errors bars represent means ± S.E. (n = 3 technical repetitions on pool of 3 mice). a, b, c: indicate a significant difference among lines (p<0.05, ANOVA).

The transgene, consisting of the mouse precursor of miR-30b (pre-miR-30b) under transcriptional control of the MMTV-LTR (Figure 2A) that is designed to drive preferential expression of the transgene in the mammary tissue [38], was micro-injected in FVB/N eggs. Thirteen founders were identified by PCR analysis of tail genomic DNA and all transmitted the transgene to their progeny. Two lines (Tg12 and Tg33) that expressed the transgene in the mammary gland were retained for further analyses. Both lines transmitted the transgene in a Mendelian fashion, indicating that both carried a single integration site. Compared analyses of these two lines allowed us to exclude a potential impact of the integration site on shared phenotype. The transgene expression was further assessed in mammary glands collected from virgin (week-6), lactating (day-12) and post-weaning (day-6) females by Northern blot (data not shown) and RT-qPCR (Figure 2B). The miR-30b expression was significantly higher in transgenic mice (Tg12 and Tg33 lines) compared to wild-type mice (p<0.05). The transgene was overexpressed 2–3 fold at the virgin stage, 15–20 fold in lactation and 6–10 fold during involution. The expression of the transgene appeared correlated with the mammary epithelial cell population. The expression was significantly higher in the Tg33 line compared to the Tg12 line at the virgin and involution stages (p<0.05), whereas no significant difference was observed in the lactation stage. The tissue-distribution of the miR-30b overexpression in these two lines was further analyzed by RT-qPCR in several tissues (liver, muscle, ovary, lung, spleen and kidney (Figure S2)). In muscle, lung and spleen, miR-30b was significantly overexpressed in transgenic mice (Tg12 and Tg33 lines) compared to wild-type mice (less than 4 fold). In liver and ovary, an organ producing important hormones for mammary gland development, no overexpression was detected. The growth and the general physiological status of the transgenic mice appeared normal.

To determine if miR-30b upregulation has an impact on the cellular miRNA biosynthesis machinery, the expression of three miRNA (let-7c, miR-26a and miR-145) and *Dicer, Drosha* and *Exportin5* were analyzed in the mammary gland of these two transgenic lines, by RT-qPCR (Figure S1). In the virgin stage, the expression of the three miRNA was not downregulated. During lactation and involution, their expression was significantly deregulated, and downregulated in some cases. In our transgenic model, expression of *Dicer*, *Drosha* and *Exportin5* was not deregulated at these stages (data not shown). Here the overexpression of a miRNA seems not caused a regulation miRNA biosynthesis process by *Dicer* expression downregulation as proposed by Bennasser and colleagues [39]. Therefore the difference of the miRNA expression observed could be due to the mammary gland phenotype.

Since the transgene was overexpressed (2–3 fold) in the mammary gland as early as the virgin stage, the induced potential phenotype was studied during all stages of adult development and differentiation of the gland. No difference in phenotype was noticed, by histology analysis, in the mammary gland collected from virgin and gestating mice, suggesting that miR-30b overexpression at these early stages has no major observable impact.

### miR-30b overexpressing mice display a mammary gland phenotype during lactation

**Figure 3:**
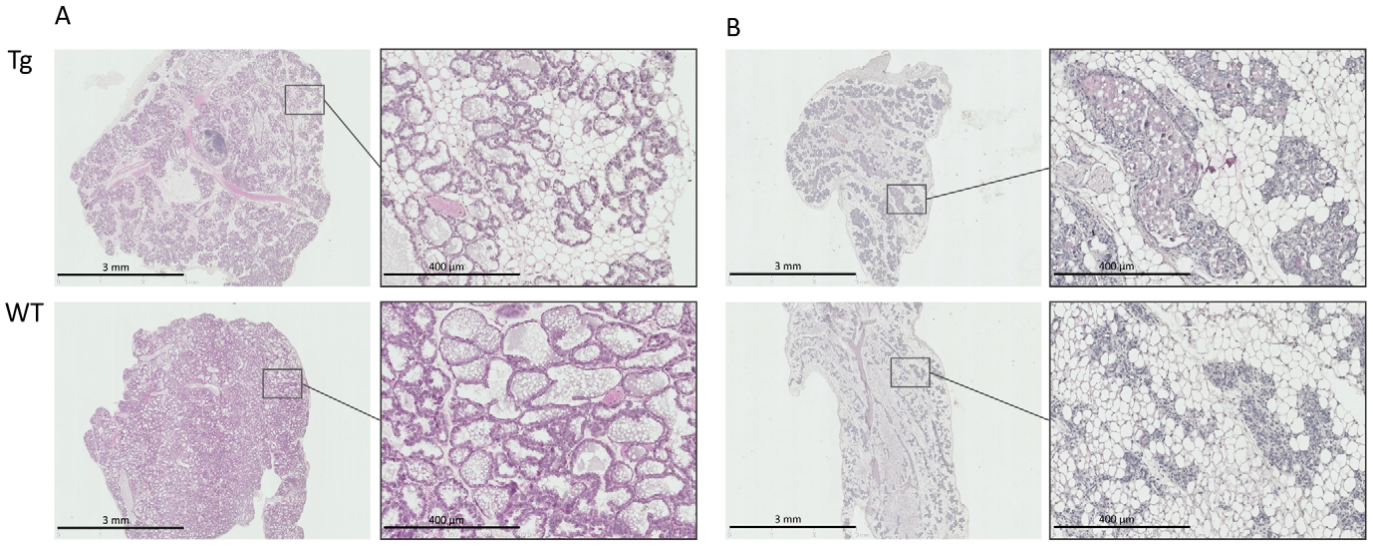
miR-30b overexpression affects the mammary gland structure during lactation and involution. Histological analyses (HES) of mammary gland from transgenic (Tg) and wild-type (WT) mice at day-12 of lactation (A) and day-6 of involution (B).

**Figure 4:**
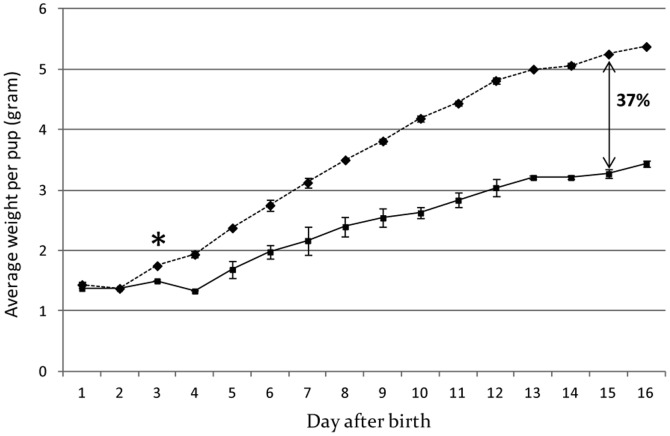
Growth of the pups is affected by overexpression of miR-30b. The weights of pups fed by dams of Tg12 line (solid line) were compared to pups fed by control wild-type mice (dotted line) from day-1 through to day-16 after birth. Here the average of weight per pup from 3 or more litters of 8 animals per dam is presented. From day-3 (*) the difference between the two curves is significantly different (p<0.05). At day-15 this difference averages 37%.

Whole-mount examination of the mammary gland from lactating (day-3 and -12) transgenic mice did not reveal any difference compared to wild-type controls. However, by histological analysis, we observed that the fat pad was less filled up with acini due to smaller lumen in transgenic mice compared to wild-type animals (Figure 3A). This phenotype was more pronounced at day-12 of lactation (Figure 3A) than at day-3 (data not shown). All phenotypes observed in Tg12 line have been also observed in Tg33 line. This mammary gland phenotype was associated with an impaired growth of the pups fed by transgenic dams compared to those suckling non-transgenic dams (Figure 4). The difference between the two growth curves was significant by day-3, and at day-15 the average body weight of pups from transgenic mothers was 37% less than controls, no lethality was observed. This impaired growth of pup persisted during the second lactation (data not shown). This phenotype could result from the high viscosity of the milk observed and the difficulty for the pups to remove the milk by suckling.

**Figure 5:**
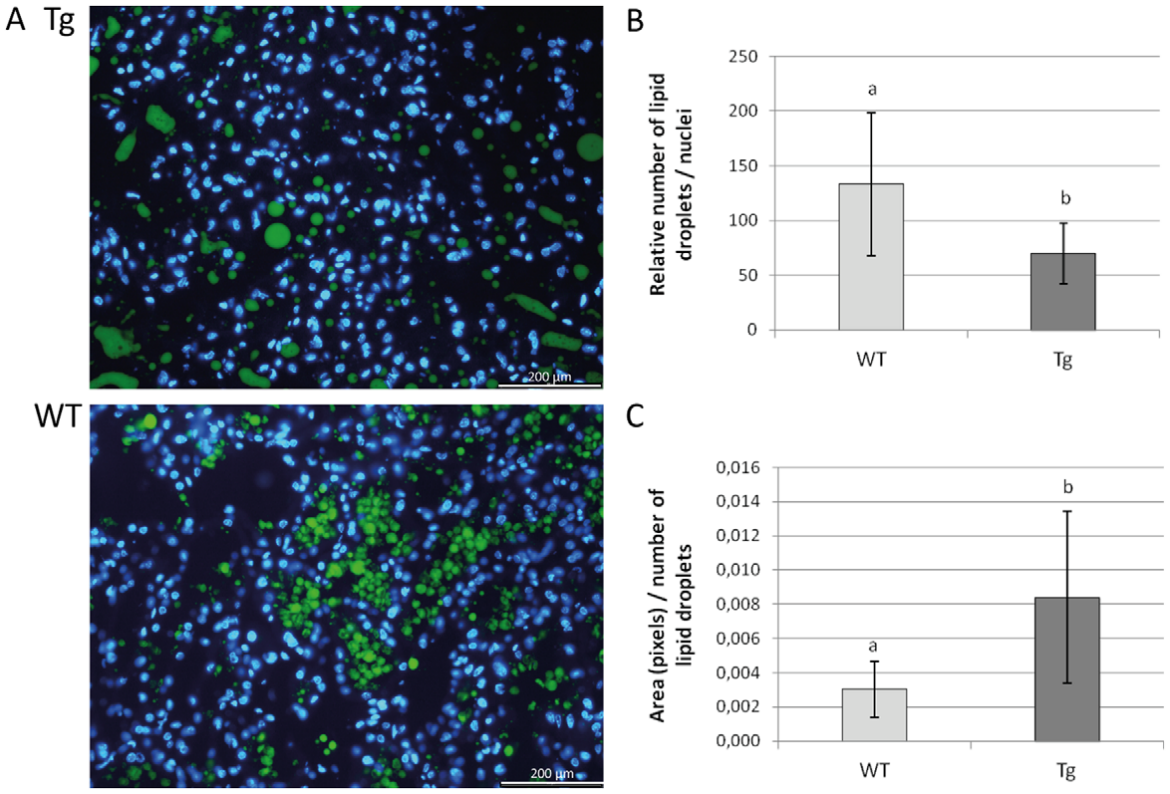
Number, size and aspect of lipid droplets are affected by overexpression of miR-30b during lactation. A/ Lipid droplets staining with Nile Red (green) and nuclei staining with DAPI (blue) on frozen histological sections of mammary gland from transgenic (Tg) and wild-type (WT) mice at day-12 of lactation. B/ The relative number of lipid droplets to number of nuclei, counted using ImageJ, is significantly lower in transgenic (Tg) than in wild-type (WT) mice. C/ The total area (measured in pixels with ImageJ) of the lipid droplets relative to their number is significantly higher in transgenic (Tg) than in wild-type (WT) mice. Data represent means ± S.E. obtained from day-12 lactating mammary glands frozen sections of 8 transgenic and 6 wild-type mice (10 pictures analyzed per individual). a, b: indicate a significant difference among lines (p<0.05, ANOVA).

We next examined the ability of alveoli of the transgenic mice to synthesize milk products including proteins and fat droplets. All major milk proteins (caseins, α-lactalbumin, β-lactoglobulin and WAP) were present in samples from transgenic mice as assessed by Coomassie staining of gels (data not shown) and the protein concentration was not different compared to wild-type controls. However, the number of lipid droplets located in the mammary tissue, detected by Red Nile staining, was smaller, however their size was bigger in transgenic compared to wild-type mice and some of these droplets lost their characteristic spherical structures (Figure 5), just as it has been observed in mammary gland of transgenic mice where Akt is constitutively expressed [40].

To determine the potential mechanisms by which overexpression of miR-30b induced this phenotype, Affymetrix Mouse Gene 1.1^ST^ arrays were performed using lactating day-12 RNA samples, prepared from 4 mice of each genotype (wild-type, lines Tg12 and Tg33) and hybridized individually. Statistical analysis of these arrays identified 220 differentially expressed genes in both transgenic lines (Tg12 and Tg33) compared to wild-type (adjusted p-value<0.05). One hundred and sixty four genes were upregulated in transgenic versus wild-type mice and 56 genes were downregulated (Table S2). Among the genes differentially expressed, 9 genes were tested for confirmation by RT-qPCR, these analysis were congruent with the microarrays data for all genes tested (Table S3).

Ingenuity Pathway Analysis (http://www.ingenuity.com/) was used to assess the top network functions associated with the overexpression of miR-30b (Table 1). All the networks were associated with tissue development, except for network 7 that was involved in the inflammatory response. Among the most overexpressed genes (Table 2), two major functions were represented: the acute phase response (*Orm2*, *Saa1*, *Saa2*, *Saa3*) and the cell adhesion (*Spp1*, *Clca2*, *Cldn4*). The importance of the deregulation of these two functions was supported by the presence of several genes involved in these functions less differentially expressed (Table S2), such as *Orm1* and *Lbp* for the acute phase response, and *Cldn1* and *Itga8* for the cell adhesion function.

**Table 1:** Ingenuity Pathway Analysis-Top Networks during lactation day-12.

ID	Top Network	Score	Genes in Network	
			*Upregulated*	*Downregulated*
**1**	Cellular Movement, Connective Tissue Development and Function, Embryonic Development	49	*Ace2, Acsl5, Agr2, Capg, Ccdc80, Cited1, Cldn1, Cldn4, Dpep1, Elf3, Htra1, Insl6, Lgals1, Lox, Mmd, Ncf2, Pdgfra, Pdlim4, Sprr2a, Tmeff1*	*Dgkz, Erbb2, Erbb4, Mlxip, Serpina5*
**2**	Antigen Presentation, Cell-To-Cell Signaling and Interaction, Hematological System Development and Function	40	*C1r, Ftl, Gsta1, Gstm1, Il18rap, Il36g, Krt19, Lbp, Mlkl, Orm1, Orm2, Pon3, Rbp4, Saa2, Serpina3, Sftpd, Tnfaip8, Tnfrsf12a, Tnfrsf22/Tnfrsf23*	*Arg2, Camk2b, Casp6*
**3**	Connective Tissue Development and Function, Skeletal and Muscular System Development and Function, Neurological Disease	31	*Arpc1b, Ccl9, Ccnb1, Cryab, Dhcr7, Hist1h2ab/Hist1h2ae, Ifi30, Ltf, Ly6a, Ly6f, Skp2, Spp1, Tmprss4, Tuba1b, Tuba1c, Tubb6*	*Itga8, Rab17*
**4**	Embryonic Development, Organ Development, Organ Morphology	29	*Dcbld2, Fam133b, Galnt12, Ldhb, Serpinb8, Snx10, Zbtb38*	*Ano4, C10orf46, Fam116a, Mia3, Mkx, Slco4c1, Ssx2ip, Susd4, Taf2*
**5**	Respiratory System Development and Function, Tissue Morphology, Hereditary Disorder	26	*Cldn1, Cystm1, Fam161a, Itm2c, Morc4, Pmf1, Pmvk, Psmg4, Srxn1*	*Atl2, Cxxc4, Dnah11, Jmy, Olfr701, Trim71*
**6**	Cellular Development, Hematological System Development and Function, Hematopoiesis	25	*Aph1b, Aph1c, Arf2, Capn6, Dennd2d, Lctl, Ly6a, Ly6f, Nup133, Pcbd1, Trim10, Zdhhc13*	*Ak4, C1orf74, C9orf152, Nat8*
**7**	Inflammatory Disease, Inflammatory Response, Skeletal and Muscular Disorders	20	*Apol6, As3mt, C7orf63, Il17re, Il36g, Kif19, Parp12, Pmm2, Spryd4*	*Ccbl1, Fam167b, Lmx1b*

**Table 2:** The most down and up regulated genes during lactation day-12.

Genes downregulated	Adjusted p-value	Fold Change
***Fmn1***	3.28E-02	−3.57
***Camk2b***	3.73E-02	−3.45
***D730048I06Rik***	3.28E-02	−3.13
***Susd4***	2.36E-02	−2.70
***Ano4***	2.93E-02	−2.22
***Limch1***	2.78E-02	−2.17
***Erbb4***	6.64E-03	−2.13
***Mkx***	1.62E-03	−2.13
***Rab17***	3.28E-02	−2.13
***Slco4c1***	1.62E-03	−2.13
***Aldh1l2***	2.65E-02	−2.08
***Arg2***	2.78E-02	−2.08
***Ak4***	2.67E-02	−2.04
***Dnahc11***	5.63E-03	−1.96
***2310057J18Rik***	3.48E-02	−1.92
**Genes upregulated**		
***Saa2***	2.12E-06	+192.07
***Saa1***	8.06E-05	+76.59
***Mir30b***	1.21E-06	+64.56
***Ly6f***	9.53E-06	+45.32
***Saa3***	4.93E-03	+17.14
***Orm2***	2.62E-02	+11.71
***Bglap2***	4.42E-05	+11.19
***Gm10573***	3.20E-02	+11.02
***Il1f9***	5.14E-06	+10.45
***9030619P08Rik***	2.78E-02	+8.55
***Bglap-rs1***	2.41E-04	+8.14
***Spp1***	1.43E-03	+7.19
***Clca2***	4.03E-02	+5.79
***Cldn4***	2.35E-02	+5.60
***Fam3c***	3.86E-03	+4.63

One of the most upregulated genes was *Ly6f*, a member of the Ly-6 family which is a group of homologous molecules linked to the outer leaflet of cell membranes via a glycophosphatidylinositol (GPI) anchor. *Ly6A* (*Sca-1*), the best characterized family member, also upregulated here, is associated with stem/progenitor cells [41] and is localized to lipid rafts where it regulates signaling complexes [42]. Recent data have shown that the knockdown of *Sca-1* in the mammary gland affects cell adhesion to a number of different extracellular matrix components [43].

The genes coding for milk proteins (WAP, casein beta…) were not differentially expressed, in agreement with the normal presence of the milk proteins (described above).

The cytoplasmic calcium concentration in mammary epithelial cells influences the size of the lipid droplets [44]. Several genes involved in calcium transport, including *Camk2b*, *Clca1*, *Clca2* and *Ano4* are strongly deregulated in the transgenic mice, potentially explaining the lipid droplet defect (Figure 5). Furthermore, *Rab17* and *Slco4c1*, which are important for the secretion and transport of molecules, are amongst the most downregulated genes. *ErbB2*, which is involved in the formation of mammary secretory alveoli, including their expansion [45] is also downexpressed, as is *ErbB4*, another member of the ErbB family that is required for the differentiation of mammary epithelial cells [45], [46], [47]. These observations could corroborate to recent published data on the miR-30 family that highlighted its role in the differentiation of various cell types including adipocytes [7], B-cells [8] or osteoblasts [9].

**Figure 6:**
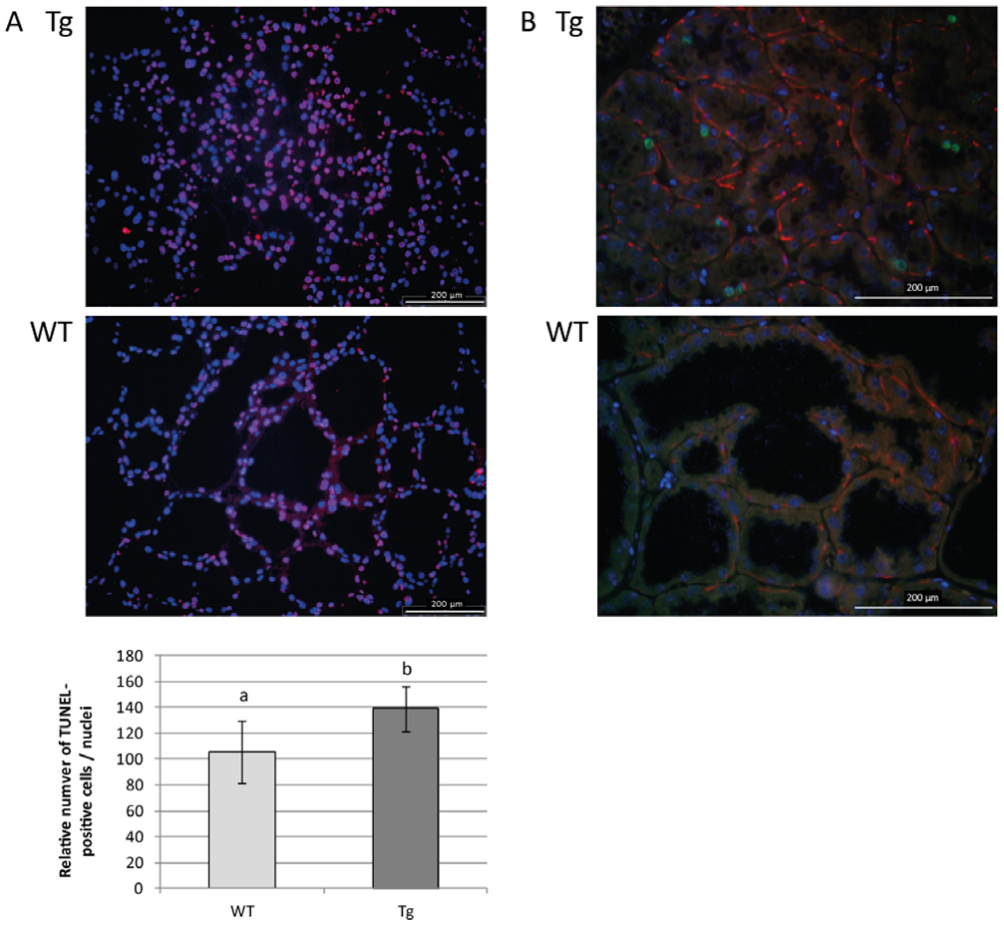
Apoptosis and proliferation processes are modified by overexpression of miR-30b during lactation. A/ Apoptosis is evaluated by TUNEL analysis: the number of TUNEL-positive cells (red) relative to the nuclei (blue) is significantly higher in transgenic (Tg) than in wild-type (WT) mice. B/ Cell proliferation activity is detected by Ki67 immunostaining: Ki67 labeled cells (green) are detected in transgenic mammary gland (Tg) samples whereas it is absent in wild-type (WT) ones. Nuclei (blue) were stained with DAPI and myoepithelial cells (red) were stained with α-SMA. Data represent means ± S.E. obtained from day-12 lactating mammary glands paraffin sections (5 pictures analyzed per individual) of 3 transgenic and 3 wild-type mice for TUNEL analysis and 6 transgenic and 7 wild-type mice for Ki67 immunostaining. a, b: indicate a significant difference among lines (p<0.05, ANOVA).

However, several differentially expressed genes we identified are known to be involved in the opposing processes of cell proliferation (downregulation of *ErbB4* [48], upregulation of *Ccnb1*), apoptosis (upregulation of *Spp1* [49], downregulation of *Arg2* [50]) and cell survey (upregulation of *Elf3*, [51]). For example, *Casp6*, a gene activated during apoptosis, was downregulated but *Saa1* that suppresses growth and accelerated apoptosis [52] was strongly overexpressed. The role of miR-30b in the proliferation or in the involution was recently demonstrated, by Ichikawa and colleagues [19] and Li and colleagues [16], respectively. The evaluation of apoptosis, by TUNEL analysis, shown a significantly increase of the number of TUNEL-positive cells in lactation (day-12) transgenic mice (Figure 6A). Moreover a low proliferative activity was detected, by Ki67 immunostaining, in transgenic lactating glands then it was absent in wild-type samples (Figure 6B). These data confirmed the transcriptomic results.

In this model, lactation could be likely maintained by a balance between the survey of the cells, their proliferation and apoptosis and an acute phase response initiated by a disruption of the structural organization due to defects of cell adhesion, secretion and transport of molecules.

Potential targets of miR-30b were searched amongst the 56 genes downregulated. None of these genes corresponds to experimentally validated target of miR-30b (Table 3). However, using a bioinformatic approach, six of them (*Limch1*, *Ano4*, *Atl2*, *Mlxip*, *Josd1*, *Itga8*) were predicted by Diana Lab algorithm (http://diana.cslab.ece.ntua.gr) to be potential targets of miR-30b. Two of these are in the list of genes the most downregulated (*Ano4* and *Limch1*). Ano4 is a member of the CaCCs (Ca^2+^-activated Cl^−^ channels), proteins involved in many important functions in cell physiology including secretion of fluids from acinar cells of secretory glands [53]. *Limch1* which is coding for a metal ion binding protein, is overexpressed in the setting of PIK3CA mutations, and such mutations have been observed in 30 to 40% of ERα-positive breast tumors [54]. If Itga8 has not been described as a crucial integrin for mammary gland development [55], [56], the importance of this family of proteins for mammary development is clearly established. The roles of the 3 other genes (*Atl2*, *Mlxip* and *Josd1*) in the mammary gland is unclear. However we found deregulated at least two genes (*Bglap2* and *Il1f9*) regulated by Runx2, a direct target of miR-30 family [7], [9]. If the level of *Runx2* mRNA is not decreased, its translation could be affected.

**Table 3:** miR-30 family targets validated in the literature.

Target	Reference	Involved in
***Bcl6***	[8]	B-cell differentiation and survey
***Ccne2***	[19]	Proliferation-cell cycle
***Ctgf***	[14]	Matrix remodelling of myocard
***Galn7, Galnt1, Sema3, Ceslr3, Twf1***	[18]	Cell invasion and immunosuppressor during metastasis
***Itgb3 and Ubc9***	[21], [62]	Apoptosis
***Lin28***	[10]	Stem cell pluripotency
***Mybl2***	[15]	Cell senescence
***Trp53***	[16]	Apoptosis
***Runx2***	[7]	Adipogenesis
***Smad1 and Runx2***	[9]	Osteogenesis
***Snail1 and Vim***	[11], [13]	EMT
***Socs1, Bcl6, Dusp10***	[63]	T-cell differentiation
***Vim***	[64]	Cell migration

### miR-30b overexpression provokes a delayed in mammary gland involution

During normal mammary gland development miR-30b expression was decreased in the early stages of involution compared to lactation (Figure 1), and thereafter increased from early to late involution. Involution is a two-step process; milk-producing alveolar cells first undergo apoptosis, and the mammary gland is then remodeled in a wave-like protease-dependent process that allows repopulation of the mammary stroma by unilocular adipocytes [57], [58]. The miR-30b upregulation was explored in mammary gland involution and tissue integrity was evaluated by whole-mount and histologic analyses. No phenotypic differences were observed by whole-mount analysis. However, a delay in involution was observed on tissue sections at day-3 and -6 after weaning (Figure 3B), which was absent at day-11 of involution (data not shown). At day-3 and -6, the mammary epithelium was maintained and some alveolar structures with open lumen persisted. However, at day-3 of involution, apical Npt2b, a marker for secreting mammary epithelial cells that is normally lost within 2 days of weaning [59], was absent in mammary glands from transgenic mice as from wild-type mice (data not shown). Moreover the number of TUNEL-positive cells was the same in transgenic and wild-type mice (data not shown). Then the delay observed in our model seems not affected the dedifferentiation of the mammary epithelial cells or the apoptosis process. However the delay of involution phenotype was not associated with mammary tumor formation during the lifespan of multiparous transgenic mice.

Affymetrix arrays were performed on involution (day-6) mammary glands from transgenic and wild-type mice. As for the lactation stage, RNA samples were prepared from mice of each genotype (4 wild-type, 4 Tg12 and 3 Tg33 mice) and these were hybridized individually. Statistical analysis revealed 1.025 differentially expressed genes in both transgenic lines (Tg12 and Tg33) compared to wild-type, and 816 were upregulated and 209 downregulated (Table S4). Nine differently-expressed genes were tested for confirmation by RT-qPCR, confirming the microarray data for all genes tested (Table S3).

Ingenuity Pathway Analysis was used to assess the top associated network functions involved in this phenotype (Table 4). The deduced networks are focused on the “inflammatory response” and the “cellular assembly/tissue morphology”. Many of the significantly deregulated genes (Tables S4 and 5) are known to play a role during the second step of involution until day-4 [60], [61], especially those coding for some chemokines (*Ccl6*, *Ccl7*, *Ccl8*, *Ccl9*), immune cell antigens (*Cd80*, *Cd86*, *Lrp1*, *Ly86*), genes involved in the acute phase response (*Saa1*, *Saa3*), genes of complement (*C1qa*, *C1qc*) or immune related genes (*Tgfb1*, *Myd88*, *Emr1*), and genes involved in cell death (*Casp1*, *Cidea*, *Phlda1*, *Tgfb1*). In addition, the overexpression of matrix metalloproteinases genes (*Mmp3/Str1, Mmp8, Mmp12, Mmp13*) and Mmp inhibitor gene (*Timp1*) indicated that the mammary gland of transgenic mice is still in full remodeling, whereas it should be more advanced on day-6 after weaning. The strong expression of genes involved in the acute phase response, particularly *Saa1* and *Saa3* (Table 5), was consistent with the inflammation expected from the observed abnormal failure to remove unnecessary differentiated alveoli. The abnormal presence of epithelial cells at this stage of involution was emphasized by a significant overexpression of *Keratin 8* (*Krt8*) and *E-cadherin* (*Cdh1*). The transcriptomic data confirmed the histological analyses on the effect of the overexpression of miR-30b on a delay of the involution. Recent data highlighted a role of miR-30 in the inhibition of EMT in hepatocytes [13], process which is important for mammary gland involution.

**Table 4:** Ingenuity Pathway Analysis-Top Networks-Involution day-6.

ID	Top Network	Score	Genes in Network	
			*Upregulated*	*Downregulated*
**1**	Cell Cycle, Cellular Assembly and Organization, DNA Replication, Recombination, and Repair	*54*	*Aurkb, Bard1, Brip1, Bub1, Calm1, Casc5, Cdc20, Cdt1, Ceacam21, Ctl1, Cpxm1, Espl1, Fxyd5, Fxyd6, Hexa, Incenp, Mxd3, Myof, Ndc80, Plxnc1, Rcn1, Sae1, Sbno2, Slc16a3, Scl39a1, Spc25, Svep1, Tgfb1, Ttk*	*Acaa2, Cpq, Epb49, Galm, Rnf152*
**2**	Cellular Compromise, Inflammatory Response, Hematological System Development and Function	*35*	*Ccl9, Cd52, Cerk, Cobl, Crlf2, Csf1, Dbnl, Flt3, Fyb, Il4r, Inpp5d, Klrap1, Kmo, Lcp1, Lcp2, Phc2, Plcg2, Plxnb2, Ptk2b, Ptprc, Sema4d, Sh3bp2, Sla, Stap1, Syn2*	*Letmd1*
**3**	Cellular Compromise, Inflammatory Response, Lipid Metabolism	*33*	*Adcy7, Ccr1, Colec12, Ctsc, Egr2, Ebp41l4a, Gjb2, Has2, Hmga1, Hn1l, Itgam, Mcl1, Mcm10, Myo1f, Naip, Pdk3, Pi4k2a, Rab31, Tnfaip8*	*Cited1, Cnst, Dlat, Parp6, Pdha1, Sox7*
**4**	Lipid Metabolism, Small Molecule Biochemistry, Tissue Morphology	*31*	*Adam8, Arid5a, Arrb2, Asah1, Ccl6, Ccr2, Ccr5, Cdh1, Cer56, Dnmt1, Gsg2, Pdpn, Pld4, Prelid1, Prkcb, Rab11fip5, Rpl13a, Slc7a11, Sptlc2, Sptssa, Tpbg, Wdr1*	*Sdhc, Sptlc3*
**5**	Hereditary Disorder, Metabolic Disease, Cellular Assembly and Organization	*29*	*Adamts4, Ap1b1, Ap1s2, B4galt5, Eno2, Eps15, Laptm5, Man1c1, Man2b1, Prtn3, Rasal1, Sap30, Sec11a, Sec11c, Serpinb1, Serpine2, Tfpi2*	*Adamts3, Ecm2, Ndufa6, Ndufs1, Ndufs7, Ndufv2*
**6**	Organ morphology, developmental Disorder, Hereditary Disorder	*27*	*Ada, Arhgap4, Arhgap9, Bst1, Ccl8, Def6, Emilin1, Ext1, Glb1, Mmp12, Muc4, Neu1, Rasgrp4, Rgs10, Sbsn, Thy1, Tnfsf8, Vcan*	*Arhgap5, Emcn, Kcnk3, Ptp4a1*
**7**	Cell Death, Cellular Function and Maintenance, Cellular Development	*27*	*Alox5, Atf3, Atp7a, Birc3, Ca9, Cd47, Eif2ak3, Evi2a, Il1rn, Il36g, Lpcat1, Msr1, Panx1, Pon2, Ripk1, Slc15a3, Tyk2*	*Cml5, Dixdc1, Fgf20, Nat8, Rorc*

**Table 5:** Genes showing the greatest degree of change at involution day-6.

Genes downregulated	Adjusted p-value	Fold Change
***Fabp3***	2.26E-02	−9.65
***Inmt***	4.25E-02	−7.25
***Cidea***	3.09E-02	−5.21
***Acot11***	4.76E-02	−4.85
***Ntrk3***	2.78E-02	−3.64
***Kcnk3***	3.69E-02	−3.45
***Tuba8***	2.95E-02	−3.27
***Ttc25***	3.21E-02	−2.94
***Clic5***	2.54E-02	−2.93
***Cited1***	2.35E-02	−2.71
***Sorcs2***	1.77E-02	−2.44
***Hadhb***	3. 11E-02	−2.33
***Fdft1***	2.46E-02	−2.33
***Dlat***	4.66E-02	−2.33
***Aco2***	3.09E-02	−2.27
**Genes upregulated**		
***miR-30b***	3.56E-03	+12.1
***Gm10573***	1.23E-02	+7.3
***Crabp1***	4.22E-02	+6.7
***Clec4e***	2.02E-02	+6.6
***Timp1***	1.47E-02	+5.5
***Saa3***	4.24E-02	+5.4
***Tm4sf19***	1.21E-02	+4.8
***Il1rn***	2.34E-02	+4.8
***Mmp8***	3.01E-02	+4.3
***Muc4***	2.16E-03	+4.2
***Saa1***	3.09E-02	+4.1
***Clec5a***	7.05E-03	+4.1
***Ccl8***	2.78E-02	+4.1
***Reg1***	4.99E-02	+4.0
***Ms4a4c***	2.14E-02	+4.0

Potential miR-30b targets genes that may be implicated in involution were searched among the 209 downregulated genes, and eight candidates (*Atl2, Fgf20, Gnao1, Meox2, Myh11, Plcb4, Ptp4a1, Ssbp2*) were identified using the Diana Lab algorithm. Some of them (*Fgf20*, *Gnao1*, *Meox2*) are involved in Wnt/β-catenin or TGFβ/Smad pathways which are important to maintain the integrity of the mammary tissue.

In conclusion, the overexpression of miR-30b causes a defect in lactation characterized by the presence of acini structures with abnormally small lumen. Even though the milk proteins are detected, the number and the structure of the lipid droplets present in the mammary tissue seems altered. Transcriptional profiling points defects in cell adhesion as well as in the secretion and transport of molecules. These phenotypes would activate a strong acute phase response and apoptosis as usually observed during involution; but sustained of the lactation seems to be assured by expression of genes involved in cell survey, proliferation and apoptosis. However the overexpression of miR-30b affects also the involution by a delay in the process.

Collectively, our findings in this novel mouse model highlight the importance of miRNA in mammary gland development, and they demonstrate that deregulation of only a single miRNA is sufficient to affect lactation and involution.

## Supporting Information

Figure S1 Expression level of let-7c, miR-26a and miR-145 in mammary gland of transgenic mice. Relative expressions of let-7c, miR-26a and miR-145 were determined by RT-qPCR in mammary gland at 3 different physiological stages (virgin, lactation and involution) in Tg12 and Tg33 lines and in control (WT) mice. miRNA expression was normalized to *U6* expression. Bars and errors bars represent means ± S.E. (n = 3 technical repetitions on pool of 3 mice). a, b, c: indicate a significant difference among lines (p<0.05, ANOVA).
(TIF)

Figure S2 miR-30b expression level in different tissues of transgenic mice. Relative expression of miR-30b was determined by RT-qPCR at different tissues (liver, muscle, ovary, lung, spleen and kidney) from the two transgenic lines (Tg12 and Tg33) and control (WT) animals. miR-30b expression was normalized to *U6* expression. Bars and errors bars represent means ± S.E. (n = 3 technical repetitions on pool of 3 mice). a, b, c: indicate a significant difference among lines (p<0.05, ANOVA).
(TIF)

Table S1 Specific primers used for RT-qPCR analysis.
(DOCX)

Table S2 List of deregulated genes in transgenic mice during lactation day-12.
(DOCX)

Table S3 Confirmation of microarray data by qRT-PCR analyses.
(DOCX)

Table S4 Deregulated genes in transgenic mice during involution day-6.
(DOCX)
