# Supplementary figures and images for: Overexpression of miR-30b in the Developing Mouse Mammary Gland Causes a Lactation Defect and Delays Involution
Source: PLoS One. 2012 Sep 24;7(9):e45727. doi: 10.1371/journal.pone.0045727 (PMC3454336; doi:10.1371/journal.pone.0045727)

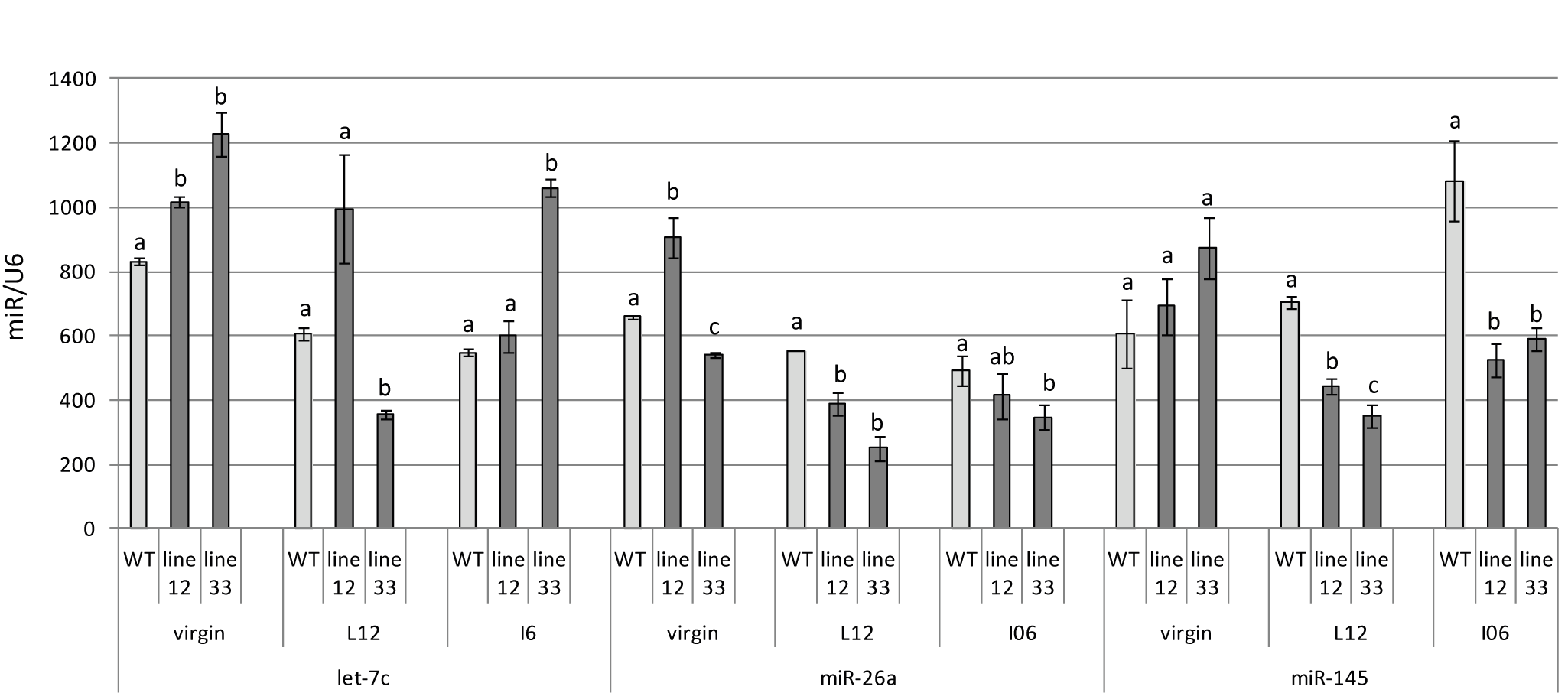

Supplement: Figure S1 — Expression level of let-7c, miR-26a and miR-145 in mammary gland of transgenic mice. Relative expressions of let-7c, miR-26a and miR-145 were determined by RT-qPCR in mammary gland at 3 different physiological stages (virgin, lactation and involution) in Tg12 and Tg33 lines and in control (WT) mice. miRNA expression was normalized to U6 expression. Bars and errors bars represent means ± S.E. (n = 3 technical repetitions on pool of 3 mice). a, b, c: indicate a significant difference among lines (p<0.05, ANOVA). (TIF) [file pone.0045727.s001.tif]

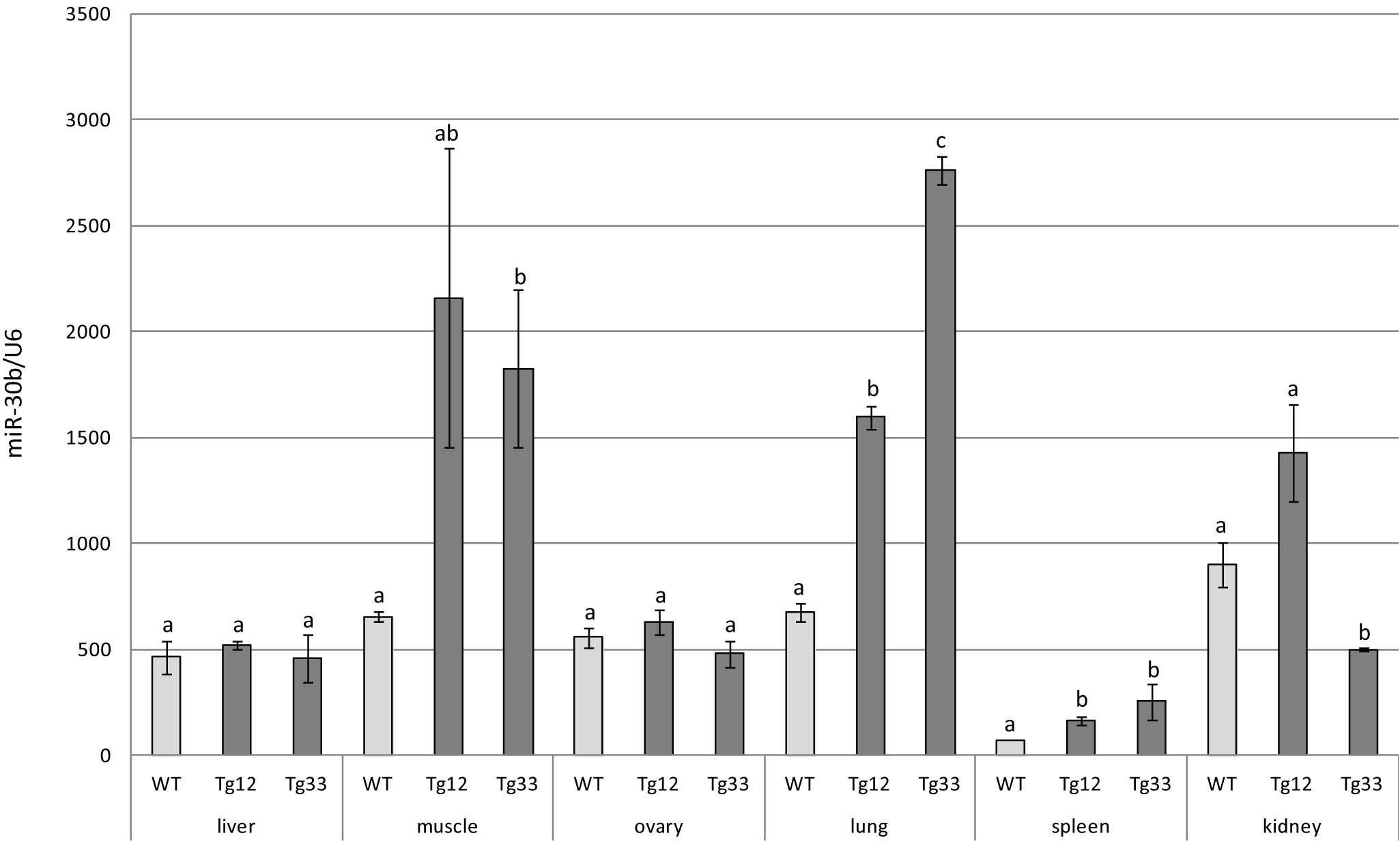

Supplement: Figure S2 — miR-30b expression level in different tissues of transgenic mice. Relative expression of miR-30b was determined by RT-qPCR at different tissues (liver, muscle, ovary, lung, spleen and kidney) from the two transgenic lines (Tg12 and Tg33) and control (WT) animals. miR-30b expression was normalized to U6 expression. Bars and errors bars represent means ± S.E. (n = 3 technical repetitions on pool of 3 mice). a, b, c: indicate a significant difference among lines (p<0.05, ANOVA). (TIF) [file pone.0045727.s002.tif]
