# Supplementary material for: Overexpression of miR-30b in the Developing Mouse Mammary Gland Causes a Lactation Defect and Delays Involution
Source: PLoS One. 2012 Sep 24;7(9):e45727. doi: 10.1371/journal.pone.0045727 (PMC3454336; doi:10.1371/journal.pone.0045727)
Supplement: Table S1 — Specific primers used for RT-qPCR analysis. (DOCX) [file pone.0045727.s003.docx]

Supplementary Table S1: Specific primers used for RT-qPCR analysis

| Gene | Sequence forward | Sequence reverse |
| --- | --- | --- |
| *Adra2b* | 5’ GACACCCCAAGCCTCCAAGGG 3’ | 5’ TCCCTTCTCTGCAGTTGCCCCA 3’ |
| *B4galt5* | 5’ CCACCCTTGCCCAGAGAGGCT 3’ | 5’ GCCCTCCGAGCTTAATGGCCG 3’ |
| *Camk2b* | 5’ GCCATCACCAGCCCCAAAGGA 3’ | 5’ CCTGGGGGCTTTGGCATCTTC 3’ |
| *Cidea* | 5’ AAGTAGCCGGCGTGGGGTGATG 3’ | 5’ CACCAGCGTAACCAGGCCAGT 3’ |
| *Cldn4* | 5’ GCCATTGAGAGCCGGTGGGC 3’ | 5’ CCCTCAAACCCGTCCATCCACT 3’ |
| *Erbb4* | 5’ CATGGCCTTCCAACATGACTCTGG 3’ | 5’ CAGCATCGGCCAGTGCAAGACT 3’ |
| *Fabp3* | 5’ AGTCACTGGTGACGCTGGACG 3’ | 5’ AGGCAGCATGGTGCTGAGCTG 3’ |
| *Fam3c* | 5’ GTGGGGAGGAGATGTGGCACC 3’ | 5’ TCAGCAATGAGCCGCCGTGC 3’ |
| *Gapdh* | 5’ CCCCAATGTGTCCGTCGTG 3’ | 5’ GCCTGCTTCACCACCTTCT 3’ |
| *Limch1* | 5’ CTGAAGGCAGCGGGACAAGGA 3’ | 5’ TCTGCTCCTCTGGCAGACCAC 3’ |
| *Lrg1* | 5’AGCCACCTTCCTGGGGTCTT 3’ | 5’AGGGCCAGGAGAAACAGGGT 3’ |
| *Ly6a* | 5’ CCAGTGCTATGGAGTCCCATTTGAG 3’ | 5’GGAGGGCAGATGGGTAAGCAAAGA 3’ |
| *Ly6f* | 5’ CTGCCTGGGAGTTTCACTTGGAAT 3’ | 5’ AGCAGGGCAGAAAGGAAAGCAG 3’ |
| *Pik3cd* | 5’ AACCTCCCCCGATCCCTGCC 3’ | 5’ AGCCCGGCCTGAACAACCAG 3’ |
| *Saa1* | 5’ CCCAGGAGACACCAGGATGAAGC 3’ | 5’ CAGTGTAGGCTCGCCACATGTCC 3’ |
| *Tbrg4* | 5’ GAGACAGAGCTGCACACGGTT 3’ | 5’ ATGTGGACCAATTTCTGGAAGGT 3’ |
