# Supplementary material for: Overexpression of miR-30b in the Developing Mouse Mammary Gland Causes a Lactation Defect and Delays Involution
Source: PLoS One. 2012 Sep 24;7(9):e45727. doi: 10.1371/journal.pone.0045727 (PMC3454336; doi:10.1371/journal.pone.0045727)
Supplement: Table S2 — List of deregulated genes in transgenic mice during lactation day-12. (DOCX) [file pone.0045727.s004.docx]

Supplementary Table S2: List of deregulated genes in transgenic mice during lactation day-12

| Gene | Affymetrix Probe Set ID | mRNA Accession | Adjusted p-value | Fold Change |
| --- | --- | --- | --- | --- |
| *Fmn1* | 10474619 | NM_010230 | 3.28E-02 | -3.57 |
| *Camk2b* | 10384064 | NM_007595 | 3.73E-02 | -3.45 |
| *D730048I06Rik* | 10592176 | NM_026593 | 3.28E-02 | -3.13 |
| *Susd4* | 10352439 | NM_144796 | 2.36E-02 | -2.70 |
| *---* | 10344582 | --- | 4.93E-03 | -2.63 |
| *---* | 10340714 | --- | 4.00E-02 | -2.33 |
| *Ano4* | 10371740 | NM_178773 | 2.93E-02 | -2.22 |
| *Limch1* | 10522217 | NM_001001980 | 2.78E-02 | -2.17 |
| *---* | 10341188 | --- | 3.36E-02 | -2.13 |
| *Erbb4* | 10355278 | NM_010154 | 6.64E-03 | -2.13 |
| *Mkx* | 10457323 | NM_177595 | 1.62E-03 | -2.13 |
| *Rab17* | 10356568 | NM_008998 | 3.28E-02 | -2.13 |
| *Slco4c1* | 10356886 | NM_172658 | 1.62E-03 | -2.13 |
| *Aldh1l2* | 10371332 | NM_153543 | 2.65E-02 | -2.08 |
| *Arg2* | 10396831 | NM_009705 | 2.78E-02 | -2.08 |
| *Ak4* | 10472923 | NM_001177602 | 2.67E-02 | -2.04 |
| *Dnahc11* | 10403112 | NM_010060 | 5.63E-03 | -1.96 |
| *---* | 10506296 | --- | 3.76E-02 | -1.92 |
| *---* | 10577217 | GENSCAN00000010055 | 2.65E-02 | -1.92 |
| *2310057J18Rik* | 10368477 | NM_026336 | 3.84E-02 | -1.92 |
| *Lmx1b* | 10481835 | NM_010725 | 3.28E-02 | -1.92 |
| *Erbb2* | 10380896 | NM_001003817 | 8.19E-03 | -1.89 |
| *Scnn1g* | 10557111 | NM_011326 | 2.78E-02 | -1.89 |
| *D630039A03Rik* | 10513190 | BC095953 | 1.36E-02 | -1.82 |
| *Nat8* | 10545877 | NM_023455 | 2.78E-02 | -1.82 |
| *Phgdh* | 10558439 | NM_016966 | 3.24E-02 | -1.82 |
| *Rnase10* | 10414533 | NM_029145 | 4.81E-02 | -1.79 |
| *---* | 10445891 | GENSCAN00000045315 | 2.78E-02 | -1.75 |
| *Slc31a1* | 10505276 | NM_175090 | 1.78E-03 | -1.75 |
| *Itga8* | 10480090 | NM_001001309 | 2.65E-02 | -1.69 |
| *2700078E11Rik* | 10468816 | NM_030197 | 2.78E-02 | -1.64 |
| *Cxxc4* | 10496182 | NM_001004367 | 2.78E-02 | -1.59 |
| *Fam167b* | 10516637 | NM_182783 | 4.42E-02 | -1.59 |
| *---* | 10338074 | --- | 4.55E-02 | -1.56 |
| *Epb4.1l4b* | 10513112 | NM_019427 | 2.65E-02 | -1.56 |
| *Ssx2ip* | 10496796 | NM_138744 | 4.88E-02 | -1.56 |
| *Drd2* | 10585169 | NM_010077 | 2.94E-02 | -1.54 |
| *Slc17a5* | 10595189 | NM_172773 | 4.33E-02 | -1.54 |
| *Trim71* | 10589909 | NM_001042503 | 2.78E-02 | -1.54 |
| *Casp6* | 10496023 | NM_009811 | 2.61E-02 | -1.52 |
| *Fam116a* | 10413282 | NM_001134465 | 3.20E-02 | -1.52 |
| *Mia3* | 10598198 | NM_177389 | 3.19E-02 | -1.52 |
| *Olfr701* | 10556020 | NM_028910 | 2.65E-02 | -1.52 |
| *---* | 10502482 | ENSMUST00000083196 | 2.67E-02 | -1.49 |
| *Cep350* | 10359050 | NM_001039184 | 3.45E-02 | -1.49 |
| *Jmy* | 10411126 | NM_021310 | 4.26E-02 | -1.49 |
| *Josd1* | 10561343 | NM_028792 | 4.88E-02 | -1.49 |
| *Serpina5* | 10398019 | NM_172953 | 4.56E-02 | -1.49 |
| *---* | 10604143 | ENSMUST00000117421 | 3.28E-02 | -1.47 |
| *Dgkz* | 10485081 | NM_001166597 | 4.88E-02 | -1.47 |
| *Sec16b* | 10350864 | NM_033354 | 4.67E-02 | -1.47 |
| *Taf2* | 10428648 | NM_001081288 | 1.62E-02 | -1.47 |
| *A130010J15Rik* | 10352829 | NM_181048 | 3.76E-02 | -1.45 |
| *Ccbl1* | 10481435 | NM_172404 | 2.94E-02 | -1.43 |
| *Atl2* | 10453062 | NM_019717 | 3.30E-02 | -1.41 |
| *Mlxip* | 10525555 | NM_133917 | 2.67E-02 | -1.41 |
| *Gca* | 10472350 | NM_145523 | 3.28E-02 | 1.34 |
| *Arpc1b* | 10604763 | NM_023142 | 2.65E-02 | 1.35 |
| *Zdhhc13* | 10553336 | NM_028031 | 4.46E-02 | 1.36 |
| *Fam133b* | 10519333 | NM_001042501 | 3.73E-02 | 1.40 |
| *Ltf* | 10589703 | NM_008522 | 2.78E-02 | 1.41 |
| *Ftl1* | 10563295 | NM_010240 | 3.19E-02 | 1.42 |
| *Pmm2* | 10433462 | NM_016881 | 3.28E-02 | 1.43 |
| *Psmg4* | 10404531 | NM_001101430 | 3.03E-02 | 1.43 |
| *Agfg2* | 10534889 | NM_178162 | 2.65E-02 | 1.44 |
| *Ftl1 // Ftl1* | 10447591 | NM_010240 | 2.78E-02 | 1.44 |
| *---* | 10477004 | ENSMUST00000122404 | 3.28E-02 | 1.45 |
| *Arpc1b* | 10527441 | NM_023142 | 7.79E-03 | 1.45 |
| *Ftl2* | 10406198 | NM_008049 | 3.20E-02 | 1.45 |
| *Tuba1c* | 10426650 | NM_009448 | 2.78E-02 | 1.45 |
| *Ftl2* | 10508069 | NM_008049 | 3.19E-02 | 1.46 |
| *---* | 10608712 | NM_138758.1 | 4.55E-02 | 1.47 |
| *Srxn1* | 10477061 | NM_029688 | 2.65E-02 | 1.48 |
| *Tuba1b* | 10432398 | NM_011654 | 2.78E-02 | 1.48 |
| *Aph1b* | 10594631 | NM_177583 | 3.10E-02 | 1.49 |
| *Ywhah* | 10494662 | NM_011738 | 3.97E-02 | 1.49 |
| *Pdgfra* | 10522503 | NM_011058 | 4.55E-02 | 1.50 |
| *Tuba1b* | 10587780 | NM_011654 | 2.62E-02 | 1.50 |
| *Insl6* | 10466932 | NM_013754 | 3.76E-02 | 1.51 |
| *Il17re* | 10540679 | NM_145826 | 3.36E-02 | 1.52 |
| *Tuba1b* | 10592058 | NM_011654 | 2.62E-02 | 1.52 |
| *Htra1* | 10558150 | NM_019564 | 3.97E-02 | 1.53 |
| *Arf2* | 10381744 | NM_007477 | 2.62E-02 | 1.54 |
| *Nup133* | 10582599 | NM_172288 | 2.35E-02 | 1.54 |
| *Spryd4* | 10373355 | NM_025716 | 4.85E-02 | 1.55 |
| *C1rb* | 10541683 | NM_001113356 | 3.03E-02 | 1.56 |
| *Itm2c* | 10347980 | NM_022417 | 3.03E-02 | 1.56 |
| *As3mt* | 10463704 | NM_020577 | 3.24E-02 | 1.57 |
| *Dhcr7* | 10559312 | NM_007856 | 3.49E-02 | 1.57 |
| *Capn6* | 10607143 | NM_007603 | 3.24E-02 | 1.59 |
| *Hebp1* | 10548761 | NM_013546 | 3.61E-02 | 1.60 |
| *Tnfaip8* | 10455647 | NM_134131 | 2.92E-02 | 1.60 |
| *0610010O12Rik* | 10454881 | BC028765 | 1.36E-02 | 1.62 |
| *Skp2* | 10427606 | NM_145468 | 2.61E-02 | 1.62 |
| *---* | 10343098 | --- | 4.83E-02 | 1.63 |
| *Dennd2d* | 10501048 | NM_001093754 | 3.74E-02 | 1.64 |
| *Lbp* | 10478048 | NM_008489 | 2.78E-02 | 1.65 |
| *Parp12* | 10544133 | NM_172893 | 3.54E-02 | 1.65 |
| *---* | 10343619 | --- | 4.88E-02 | 1.66 |
| *Penk* | 10511363 | NM_001002927 | 2.94E-02 | 1.66 |
| *Dcbld2* | 10436372 | NM_028523 | 3.45E-02 | 1.70 |
| *Acsl5* | 10464045 | NM_027976 | 2.62E-02 | 1.71 |
| *Carhsp1* | 10497752 | NM_025821 | 3.97E-02 | 1.72 |
| *2610029I01Rik* | 10511803 | NM_029840 | 3.54E-02 | 1.76 |
| *Cryab* | 10585214 | NM_009964 | 2.67E-02 | 1.76 |
| *Dpep1* | 10576235 | NM_007876 | 2.78E-02 | 1.76 |
| *Sh3d20* | 10391914 | NM_183288 | 2.78E-02 | 1.76 |
| *Pdlim4* | 10385903 | NM_019417 | 2.61E-02 | 1.77 |
| *Stom* | 10482030 | NM_013515 | 2.78E-02 | 1.77 |
| *Lox* | 10458894 | NM_010728 | 4.21E-02 | 1.78 |
| *Mlkl* | 10581813 | NM_029005 | 2.78E-02 | 1.78 |
| *Pmf1* | 10499366 | NM_025928 | 3.28E-02 | 1.78 |
| *Kif19a* | 10382409 | NM_001102615 | 3.84E-02 | 1.79 |
| *Tmeff1* | 10504891 | NM_021436 | 4.03E-02 | 1.79 |
| *---* | 10339231 | --- | 4.55E-02 | 1.81 |
| *Pmvk* | 10493548 | NM_026784 | 2.65E-02 | 1.82 |
| *Lgals1* | 10425161 | NM_008495 | 3.19E-02 | 1.85 |
| *Ifi30* | 10579347 | NM_023065 | 3.03E-02 | 1.87 |
| *BC049349* | 10572733 | NM_001164581 | 2.62E-02 | 1.88 |
| *Agr2* | 10395365 | NM_011783 | 4.52E-02 | 1.90 |
| *Tubb6* | 10456400 | NM_026473 | 2.82E-02 | 1.91 |
| *Pcbd1* | 10363455 | NM_025273 | 4.93E-03 | 1.92 |
| *Gstm2* | 10501222 | NM_008183 | 5.00E-03 | 1.93 |
| *Ldhb* | 10549097 | NM_008492 | 4.88E-02 | 1.94 |
| *Morc4* | 10606948 | NM_029413 | 2.78E-02 | 1.94 |
| *Snx10* | 10538290 | NM_028035 | 2.94E-02 | 1.94 |
| *---* | 10342955 | --- | 2.78E-02 | 1.96 |
| *Ldhb* | 10462035 | NM_008492 | 3.54E-02 | 1.97 |
| *Ly6a* | 10429564 | NM_010738 | 2.65E-02 | 1.97 |
| *Sftpd* | 10419096 | NM_009160 | 3.05E-02 | 1.99 |
| *Trim10* | 10445046 | NM_011280 | 2.65E-02 | 1.99 |
| *Cited1* | 10606083 | NM_007709 | 2.94E-02 | 2.01 |
| *Il18rap* | 10345824 | NM_010553 | 2.82E-02 | 2.01 |
| *---* | 10339290 | --- | 2.78E-02 | 2.03 |
| *Elf3* | 10358027 | NM_001163131 | 4.42E-02 | 2.05 |
| *Ccdc80* | 10435948 | NM_026439 | 1.36E-02 | 2.06 |
| *Ccl9* | 10389214 | NM_011338 | 2.97E-02 | 2.06 |
| *H2-gs10* | 10444814 | NM_001143689 | 2.67E-02 | 2.06 |
| *Capg* | 10539135 | NM_007599 | 3.28E-02 | 2.08 |
| *Aph1c* | 10594638 | NM_026674 | 3.03E-02 | 2.09 |
| *Ace2* | 10603066 | NM_027286 | 4.03E-02 | 2.10 |
| *Dusp8* | 10569280 | NM_008748 | 2.67E-02 | 2.11 |
| *Fam161a* | 10374578 | NM_028672 | 3.36E-02 | 2.12 |
| *Mcm4* | 10437945 | NM_008565 | 4.46E-02 | 2.12 |
| *Tshr* | 10397606 | NM_011648 | 3.28E-02 | 2.12 |
| *Ckmt1* | 10475324 | NM_009897 | 4.21E-02 | 2.13 |
| *Sprr2a1* | 10493850 | NM_011468 | 2.65E-02 | 2.13 |
| *Cysltr1* | 10606355 | NM_021476 | 3.19E-02 | 2.15 |
| *Ctsz* | 10490212 | NM_022325 | 5.00E-03 | 2.18 |
| *Rbp4* | 10467319 | NM_001159487 | 3.49E-02 | 2.23 |
| *Tnfrsf12a* | 10448307 | NM_013749 | 4.21E-02 | 2.35 |
| *Ggt5* | 10364072 | NM_011820 | 1.62E-02 | 2.37 |
| *---* | 10340680 | --- | 1.74E-02 | 2.39 |
| *BC018473* | 10382846 | NR_003364 | 2.78E-02 | 2.39 |
| *Pon3* | 10542993 | NM_173006 | 1.36E-02 | 2.42 |
| *Hist1h2ab* | 10404063 | NM_175660 | 4.88E-02 | 2.47 |
| *Gm5458* | 10417492 | NM_001024706 | 3.19E-02 | 2.52 |
| *Mmd* | 10380289 | NM_026178 | 3.36E-02 | 2.55 |
| *Myl1* | 10355259 | NM_021285 | 2.36E-02 | 2.58 |
| *Serpina3n* | 10398075 | NM_009252 | 6.64E-03 | 2.58 |
| *Gm1973* | 10417415 | NM_029288 | 3.24E-02 | 2.71 |
| *Gm1973* | 10412513 | NM_029288 | 2.94E-02 | 2.74 |
| *Galnt12* | 10504763 | NM_172693 | 2.78E-02 | 2.75 |
| *A330021E22Rik* | 10527982 | BC062906 | 8.19E-03 | 2.77 |
| *Tmprss4* | 10593035 | NM_145403 | 6.27E-03 | 2.78 |
| *Krt19* | 10391036 | NM_008471 | 2.65E-02 | 2.81 |
| *Gm1973* | 10412543 | NM_029288 | 2.78E-02 | 2.89 |
| *Gm1973* | 10417504 | NM_029288 | 3.76E-02 | 2.91 |
| *Lctl* | 10586140 | NM_145835 | 4.68E-02 | 2.91 |
| *---* | 10412503 | GENSCAN00000009258 | 2.94E-02 | 2.94 |
| *Gm5458* | 10417773 | NM_001024706 | 4.14E-02 | 2.95 |
| *Gsta3* | 10345065 | NM_001077353 | 1.65E-02 | 2.96 |
| *Ccnb1* | 10411739 | NM_172301 | 3.14E-02 | 3.00 |
| *Gm1973* | 10417253 | NM_029288 | 2.65E-02 | 3.03 |
| *Gm1973* | 10417281 | NM_029288 | 2.65E-02 | 3.03 |
| *Gm1973* | 10417239 | NM_029288 | 4.68E-02 | 3.05 |
| *Gm3002* | 10417359 | NR_033388 | 4.46E-02 | 3.06 |
| *Ccnb1* | 10562637 | NM_172301 | 3.03E-02 | 3.07 |
| *Ccnb1* | 10515836 | NM_172301 | 3.24E-02 | 3.10 |
| *Gm3002* | 10417302 | NR_033388 | 4.27E-02 | 3.13 |
| *Gm3002* | 10417411 | NR_033388 | 3.76E-02 | 3.29 |
| *Gm3002* | 10417258 | NR_033388 | 3.28E-02 | 3.37 |
| *Gm5458* | 10417501 | NM_001024706 | 2.82E-02 | 3.37 |
| *Serpinb8* | 10349174 | NM_011459 | 2.78E-02 | 3.37 |
| *Apol6* | 10425031 | NM_028010 | 2.65E-02 | 3.40 |
| *Slc16a4* | 10495206 | NM_146136 | 1.13E-03 | 3.41 |
| *Aox3* | 10346410 | NM_023617 | 4.00E-02 | 3.43 |
| *---* | 10338931 | --- | 4.88E-02 | 3.44 |
| *Gm2897* | 10417235 | NM_001177714 | 3.19E-02 | 3.46 |
| *Gm2897* | 10417315 | NM_001177714 | 3.19E-02 | 3.46 |
| *Gm5458* | 10417458 | NM_001024706 | 2.79E-02 | 3.51 |
| *Cldn1* | 10438769 | NM_016674 | 3.49E-02 | 3.56 |
| *Zbtb38* | 10595836 | ENSMUST00000152594 | 2.78E-02 | 3.60 |
| *Tmem82* | 10517980 | NM_145987 | 3.09E-04 | 3.61 |
| *Tnfrsf23* | 10569504 | NM_024290 | 9.81E-03 | 3.65 |
| *Gm1973* | 10417245 | NM_029288 | 2.62E-02 | 3.67 |
| *Ncf2* | 10412123 | NM_010877 | 3.86E-03 | 3.67 |
| *Pkd2l1* | 10467962 | NM_181422 | 3.24E-02 | 3.72 |
| *D830030K20Rik* | 10412549 | NM_177135 | 2.35E-02 | 3.82 |
| *D830030K20Rik* | 10417319 | NM_177135 | 2.65E-02 | 3.88 |
| *D830030K20Rik* | 10417408 | NM_177135 | 1.36E-02 | 4.15 |
| *Orm1* | 10505438 | NM_008768 | 4.93E-03 | 4.45 |
| *Dpyd* | 10495625 | NM_170778 | 2.65E-02 | 4.54 |
| *Olfr1328* | 10515834 | NM_146399 | 1.03E-02 | 4.61 |
| *Fam3c* | 10543319 | NM_138587 | 3.86E-03 | 4.63 |
| *Cldn4* | 10534395 | NM_009903 | 2.35E-02 | 5.60 |
| *Clca2* | 10502565 | NM_030601 | 4.03E-02 | 5.79 |
| *Spp1* | 10523717 | NM_009263 | 1.43E-03 | 7.19 |
| *---* | 10524980 | ENSMUST00000119191 | 1.29E-03 | 7.55 |
| *Bglap-rs1* | 10499354 | NM_031368 | 2.41E-04 | 8.14 |
| *9030619P08Rik* | 10429588 | NM_001039720 | 2.78E-02 | 8.55 |
| *Il1f9* | 10469786 | NM_153511 | 5.14E-06 | 10.45 |
| *Gm10573* | 10516042 | ENSMUST00000097904 | 3.20E-02 | 11.02 |
| *Bglap2* | 10499358 | NM_001032298 | 4.42E-05 | 11.19 |
| *Orm2* | 10505451 | NM_011016 | 2.62E-02 | 11.71 |
| *Saa3* | 10563597 | NM_011315 | 4.93E-03 | 17.14 |
| *Ly6f* | 10424691 | NM_008530 | 9.53E-06 | 45.32 |
| *Mir30b* | 10429197 | NR_029534 | 1.21E-06 | 64.56 |
| *Saa1* | 10563611 | NM_009117 | 8.06E-05 | 76.59 |
| *Saa2* | 10553274 | NM_011314 | 2.12E-06 | 192.07 |
