# Supplementary material for: Overexpression of miR-30b in the Developing Mouse Mammary Gland Causes a Lactation Defect and Delays Involution
Source: PLoS One. 2012 Sep 24;7(9):e45727. doi: 10.1371/journal.pone.0045727 (PMC3454336; doi:10.1371/journal.pone.0045727)
Supplement: Table S3 — Confirmation of microarray data by qRT-PCR analyses. (DOCX) [file pone.0045727.s005.docx]

Supplementary Table S3: Confirmation of microarray data by qRT-PCR analyses

| Gene | FC Lactation day-12 | | FC Involution day-06 | |
| --- | --- | --- | --- | --- |
|  | Affymetrix® array | qPCR | Affymetrix® array | qPCR |
| *Adra2b* | -1.6 | -1.1 | *ns* | *nd* |
| *B4galt5* | *ns* | *nd* | +2.1 | +3.3 |
| *Camk2b* | -3.5 | -36.8 | -1.5 | -1.2 |
| *Cidea* | *ns* | *nd* | -5.3 | -1.8 |
| *Cldn4* | +5.6 | +38.1 | +3.2 | +6.2 |
| *Erbb4* | - 2.1 | -5.8 | *ns* | *nd* |
| *Fabp3* | *ns* | *nd* | -9.2 | *-*2.8 |
| *Fam3c* | +4.6 | +10.9 | +2.5 | +4.8 |
| *Limch1* | -2.2 | -4.6 | *ns* | *nd* |
| *Lrg1* | *ns* | *nd* | +2.5 | +1.9 |
| *Ly6a* | +2.0 | +8.1 | *ns* | *nd* |
| *Ly6f* | +45.3 | +1.8E02 | *ns* | *nd* |
| *Pik3cd* | *ns* | *nd* | +1.5 | +3.5 |
| *Saa1* | +76.6 | +1.1E04 | +4.1 | +5.7 |

FC: Fold change

ns: transgenic and wild-type mice are non significantly different with adjusted p-value ≤ 0.05

nd: not determined
