# Supplementary material for: Overexpression of miR-30b in the Developing Mouse Mammary Gland Causes a Lactation Defect and Delays Involution
Source: PLoS One. 2012 Sep 24;7(9):e45727. doi: 10.1371/journal.pone.0045727 (PMC3454336; doi:10.1371/journal.pone.0045727)
Supplement: Table S4 — Deregulated genes in transgenic mice during involution day-6. (DOCX) [file pone.0045727.s006.docx]

Supplementary Table S4: Deregulated genes in transgenic mice during involution day-6

| Gene | Affymetrix Probe Set ID | mRNA Accession | Adjusted p-value | Fold Change |
| --- | --- | --- | --- | --- |
| *Fabp3* | 10508614 | NM_010174 | 2.33E-02 | -10.00 |
| *Fabp3* | 10371502 | NM_010174 | 2.19E-02 | -8.33 |
| *Inmt* | 10544932 | NM_009349 | 4.25E-02 | -7.14 |
| *Cidea* | 10456392 | NM_007702 | 3.09E-02 | -5.26 |
| *Acot11* | 10514865 | NM_025590 | 4.76E-02 | -4.76 |
| *Ntrk3* | 10564667 | NM_008746 | 2.78E-02 | -3.70 |
| *Kcnk3* | 10520506 | NM_010608 | 3.69E-02 | -3.45 |
| *Tuba8* | 10541301 | NM_017379 | 2.95E-02 | -3.23 |
| *Ttc25* | 10381140 | NM_028918 | 3.21E-02 | -2.94 |
| *Clic5* | 10445347 | NM_172621 | 2.54E-02 | -2.94 |
| *Cited1* | 10606083 | NM_007709 | 2.35E-02 | -2.70 |
| *---* | 10341739 | --- | 4.74E-02 | -2.63 |
| *---* | 10344622 | GENSCAN00000047522 | 4.89E-02 | -2.56 |
| *Sorcs2* | 10529515 | NM_030889 | 1.77E-02 | -2.44 |
| *Hadhb* | 10520467 | NM_145558 | 3.13E-02 | -2.33 |
| *Fdft1* | 10412909 | NM_010191 | 2.46E-02 | -2.33 |
| *Hadhb* | 10474379 | NM_145558 | 3.10E-02 | -2.33 |
| *Dlat* | 10593367 | NM_145614 | 4.66E-02 | -2.33 |
| *Aco2* | 10425611 | NM_080633 | 3.09E-02 | -2.27 |
| *Letmd1* | 10426909 | NM_134093 | 4.50E-02 | -2.27 |
| *Pdha1* | 10607587 | NM_008810 | 3.09E-02 | -2.22 |
| *Gnao1* | 10573979 | NM_010308 | 2.95E-02 | -2.22 |
| *---* | 10338660 | --- | 2.77E-02 | -2.13 |
| *Fdft1* | 10420730 | NM_010191 | 2.90E-02 | -2.13 |
| *Pm20d1* | 10349694 | NM_178079 | 4.68E-02 | -2.13 |
| *Cnst* | 10352166 | NM_146105 | 3.11E-02 | -2.13 |
| *Inca1* | 10387971 | NM_213729 | 3.47E-02 | -2.08 |
| *Cyc1* | 10424825 | NM_025567 | 3.44E-02 | -2.08 |
| *Apoo* | 10412036 | NM_026673 | 3.40E-02 | -2.08 |
| *---* | 10569456 | ENSMUST00000083184 | 2.96E-02 | -2.08 |
| *Fn3k* | 10383564 | NM_001038699 | 3.72E-02 | -2.04 |
| *Apoo* | 10600797 | NM_026673 | 3.92E-02 | -2.00 |
| *Ccdc3* | 10469066 | NM_028804 | 1.81E-02 | -2.00 |
| *Aldoart1* | 10513912 | EF662061 | 1.28E-02 | -1.96 |
| *---* | 10343036 | --- | 2.89E-02 | -1.96 |
| *Rasgef1c* | 10375650 | NM_029004 | 2.95E-02 | -1.96 |
| *Ndufs1* | 10355084 | NM_001160038 | 4.42E-02 | -1.92 |
| *Tcfap2b* | 10345016 | NM_009334 | 2.48E-02 | -1.92 |
| *1810010H24Rik* | 10382136 | NM_001163473 | 2.76E-02 | -1.92 |
| *---* | 10338813 | --- | 2.95E-02 | -1.92 |
| *Dbt* | 10495549 | NM_010022 | 4.53E-02 | -1.92 |
| *---* | 10407022 | ENSMUST00000095488 | 1.88E-02 | -1.92 |
| *Me3* | 10554819 | NM_181407 | 2.95E-02 | -1.92 |
| *---* | 10339603 | --- | 3.11E-02 | -1.92 |
| *Sctr* | 10349319 | NM_001012322 | 3.86E-02 | -1.89 |
| *Epb4.9* | 10421418 | NM_013514 | 4.99E-02 | -1.89 |
| *---* | 10341313 | --- | 4.53E-02 | -1.89 |
| *Cs* | 10367292 | NM_026444 | 3.96E-02 | -1.85 |
| *Ddo* | 10362701 | NM_027442 | 2.85E-02 | -1.85 |
| *Acaa2* | 10456699 | NM_177470 | 3.21E-02 | -1.85 |
| *Gstm1* | 10501229 | NM_010358 | 3.09E-02 | -1.85 |
| *Rnf152* | 10357003 | NM_178779 | 2.61E-02 | -1.85 |
| *Dlst* | 10397311 | NM_030225 | 4.66E-02 | -1.85 |
| *---* | 10339526 | --- | 3.77E-02 | -1.85 |
| *Pik3c2g* | 10542477 | NM_207683 | 2.44E-02 | -1.85 |
| *Uqcrc1* | 10589196 | NM_025407 | 3.19E-02 | -1.82 |
| *Fitm1* | 10415279 | NM_026808 | 2.34E-02 | -1.82 |
| *Mdk* | 10485070 | NM_010784 | 3.64E-02 | -1.82 |
| *Phyh* | 10469046 | NM_010726 | 3.31E-02 | -1.79 |
| *Agl* | 10501699 | NM_001081326 | 4.39E-02 | -1.79 |
| *Oscp1* | 10508099 | NM_172701 | 3.86E-02 | -1.79 |
| *---* | 10340757 | --- | 3.98E-02 | -1.79 |
| *2900006K08Rik* | 10397230 | NM_028377 | 2.85E-02 | -1.79 |
| *Dixdc1* | 10593384 | NM_178118 | 3.99E-02 | -1.79 |
| *Mdk // Mdk* | 10381601 | AY864926 | 3.98E-02 | -1.79 |
| *Cml5* | 10545874 | NM_023493 | 3.20E-02 | -1.75 |
| *---* | 10433177 | GENSCAN00000043930 | 4.86E-02 | -1.75 |
| *Cacnb4* | 10482695 | NM_001037099 | 3.46E-02 | -1.75 |
| *---* | 10340318 | --- | 1.29E-02 | -1.72 |
| *Nat8* | 10545877 | NM_023455 | 3.31E-02 | -1.72 |
| *Galm* | 10447084 | NM_176963 | 3.21E-02 | -1.72 |
| *Prkaa2* | 10514779 | NM_178143 | 1.28E-02 | -1.72 |
| *Tmem135* | 10565437 | NM_028343 | 3.80E-02 | -1.72 |
| *Sox7* | 10415885 | NM_011446 | 2.95E-02 | -1.72 |
| *Ech1* | 10551614 | NM_016772 | 4.41E-02 | -1.69 |
| *Csl // Csl* | 10372116 | NM_027945 | 2.83E-02 | -1.69 |
| *Phb* | 10422776 | NM_008831 | 3.35E-02 | -1.69 |
| *Zfp354b* | 10385665 | NM_013744 | 2.85E-02 | -1.69 |
| *---* | 10339244 | --- | 1.36E-02 | -1.69 |
| *Phb* | 10380551 | NM_008831 | 3.31E-02 | -1.69 |
| *Tecr* | 10579996 | NM_134118 | 4.93E-02 | -1.69 |
| *Rorc* | 10494023 | NM_011281 | 4.83E-02 | -1.69 |
| *Myh11* | 10437885 | NM_013607 | 3.40E-02 | -1.67 |
| *---* | 10366705 | GENSCAN00000002143 | 4.79E-02 | -1.67 |
| *Grik5* | 10560945 | NM_008168 | 2.48E-02 | -1.67 |
| *Ak4* | 10472923 | NM_001177602 | 4.39E-02 | -1.67 |
| *Ecm2* | 10405033 | NM_001012324 | 4.49E-02 | -1.67 |
| *Plcb4* | 10476443 | NM_013829 | 3.74E-02 | -1.67 |
| *Sdr39u1* | 10420216 | NM_001082975 | 4.60E-02 | -1.67 |
| *4933403F05Rik* | 10459604 | NM_153794 | 3.69E-02 | -1.67 |
| *---* | 10551529 | ENSMUST00000075698 | 3.00E-02 | -1.64 |
| *Acsf3* | 10576160 | NM_144932 | 4.84E-02 | -1.64 |
| *Sdhc* | 10360046 | NM_025321 | 4.76E-02 | -1.64 |
| *---* | 10400892 | GENSCAN00000047200 | 1.82E-02 | -1.64 |
| *Gm13363* | 10469575 | NR_002688 | 2.02E-02 | -1.64 |
| *---* | 10608676 | NM_145443.1 | 3.47E-02 | -1.64 |
| *---* | 10362672 | ENSMUST00000099956 | 4.03E-02 | -1.64 |
| *Aldoa* | 10568050 | NM_001177307 | 4.97E-02 | -1.64 |
| *Slc40a1* | 10354374 | NM_016917 | 3.22E-02 | -1.61 |
| *Itga1* | 10412298 | NM_001033228 | 4.75E-02 | -1.61 |
| *Gstm3* | 10362511 | NM_010359 | 3.03E-02 | -1.61 |
| *Sptlc3* | 10476545 | NM_175467 | 2.39E-02 | -1.61 |
| *---* | 10344521 | --- | 1.41E-02 | -1.61 |
| *---* | 10364091 | GENSCAN00000005172 | 4.84E-02 | -1.59 |
| *Ptp4a1* | 10463068 | NM_011200 | 2.48E-02 | -1.59 |
| *Dpf3* | 10401359 | NM_058212 | 4.87E-02 | -1.59 |
| *Ptp4a1* | 10548563 | NM_011200 | 2.48E-02 | -1.59 |
| *Mmachc* | 10515363 | NM_025962 | 4.29E-02 | -1.59 |
| *Ptp4a1* | 10353707 | NM_011200 | 2.85E-02 | -1.59 |
| *Gpi1* | 10570000 | NM_008155 | 4.75E-02 | -1.59 |
| *Filip1* | 10595298 | NM_001081243 | 1.28E-02 | -1.59 |
| *---* | 10341200 | --- | 4.25E-02 | -1.56 |
| *Ptp4a1* | 10467489 | NM_011200 | 2.93E-02 | -1.56 |
| *Tbc1d7* | 10408870 | NM_025935 | 1.74E-02 | -1.56 |
| *Eno1* | 10473240 | NM_023119 | 4.75E-02 | -1.56 |
| *Adamts3* | 10531179 | NM_177872 | 4.86E-02 | -1.56 |
| *---* | 10507872 | ENSMUST00000083844 | 3.09E-02 | -1.56 |
| *Rbm24* | 10404904 | NM_001081425 | 3.46E-02 | -1.56 |
| *Ghitm* | 10419038 | NM_078478 | 4.58E-02 | -1.54 |
| *---* | 10343603 | --- | 3.52E-02 | -1.54 |
| *Eno1* | 10510546 | NM_023119 | 3.54E-02 | -1.54 |
| *4930578C19Rik* | 10603796 | BC118515 | 2.95E-02 | -1.54 |
| *Ugp2* | 10384579 | NM_139297 | 2.38E-02 | -1.54 |
| *Plekhh3* | 10391359 | NM_146030 | 2.36E-02 | -1.54 |
| *Isoc2b* | 10559687 | NM_026158 | 3.98E-02 | -1.54 |
| *Ndufv2* | 10452525 | NM_028388 | 4.53E-02 | -1.54 |
| *Bak1* | 10443117 | NM_007523 | 2.78E-02 | -1.54 |
| *Eno1* | 10455595 | NM_023119 | 4.84E-02 | -1.54 |
| *Ndufs7* | 10364744 | NM_029272 | 4.47E-02 | -1.54 |
| *---* | 10344519 | --- | 3.96E-02 | -1.52 |
| *---* | 10343580 | --- | 2.97E-02 | -1.52 |
| *---* | 10340866 | --- | 2.95E-02 | -1.49 |
| *Ndufa6* | 10430846 | NM_025987 | 2.93E-02 | -1.49 |
| *Camk2b* | 10384064 | NM_007595 | 4.91E-02 | -1.49 |
| *Mkrn2* | 10540923 | NM_023290 | 3.51E-02 | -1.49 |
| *---* | 10507870 | ENSMUST00000082721 | 3.76E-02 | -1.49 |
| *P2ry1* | 10492330 | NM_008772 | 2.90E-02 | -1.49 |
| *---* | 10338496 | --- | 2.78E-02 | -1.47 |
| *---* | 10374313 | ENSMUST00000117538 | 2.19E-02 | -1.47 |
| *Emcn* | 10496359 | NM_001163522 | 3.69E-02 | -1.47 |
| *Tubg1* | 10381250 | NM_134024 | 2.72E-02 | -1.47 |
| *Hepacam2* | 10542917 | NM_178899 | 4.06E-02 | -1.47 |
| *---* | 10364383 | GENSCAN00000014373 | 2.90E-02 | -1.47 |
| *2700089E24Rik* | 10542264 | NM_001163445 | 3.14E-02 | -1.47 |
| *Meox2* | 10395409 | NM_008584 | 4.75E-02 | -1.47 |
| *Nicn1* | 10588876 | NM_025449 | 1.83E-02 | -1.45 |
| *D0H4S114* | 10458046 | NM_053078 | 3.14E-02 | -1.45 |
| *---* | 10504916 | ENSMUST00000117128 | 2.76E-02 | -1.45 |
| *Nckap5* | 10357345 | NM_172484 | 2.98E-02 | -1.45 |
| *---* | 10583806 | GENSCAN00000046181 | 4.07E-02 | -1.45 |
| *Gm5069* | 10360662 | NR_003623 | 3.31E-02 | -1.45 |
| *Tet1* | 10369702 | NM_027384 | 3.14E-02 | -1.43 |
| *Zfp189* | 10504918 | NM_145547 | 2.93E-02 | -1.43 |
| *Rgnef* | 10411395 | NM_012026 | 3.21E-02 | -1.43 |
| *Gm10291 // Gm10291* | 10386947 | ENSMUST00000094175 | 3.19E-02 | -1.43 |
| *---* | 10414431 | AK188366 | 2.35E-02 | -1.43 |
| *---* | 10582821 | ENSMUST00000083703 | 3.69E-02 | -1.43 |
| *Gucy1a2* | 10582958 | NM_001033322 | 2.33E-02 | -1.43 |
| *Kif1b* | 10518585 | NM_207682 | 3.09E-02 | -1.43 |
| *Clec1a* | 10548367 | NM_175526 | 3.46E-02 | -1.43 |
| *Stox2* | 10578572 | NM_001114311 | 2.38E-02 | -1.43 |
| *Atoh8* | 10545372 | NM_153778 | 4.84E-02 | -1.43 |
| *Rbbp9* | 10488291 | NM_015754 | 2.35E-02 | -1.43 |
| *---* | 10341788 | --- | 1.58E-02 | -1.43 |
| *Zmat1* | 10606770 | NM_175446 | 4.66E-02 | -1.41 |
| *Gm10291* | 10554817 | ENSMUST00000094175 | 2.85E-02 | -1.41 |
| *---* | 10580984 | GENSCAN00000045727 | 4.74E-02 | -1.41 |
| *---* | 10417700 | GENSCAN00000049061 | 4.34E-02 | -1.41 |
| *Pcdhb6* | 10455069 | NM_053131 | 2.33E-02 | -1.41 |
| *---* | 10482432 | ENSMUST00000098560 | 2.90E-02 | -1.41 |
| *Scoc* | 10579987 | NM_001039137 | 2.60E-02 | -1.41 |
| *Ptcd3* | 10545346 | NM_027275 | 4.79E-02 | -1.41 |
| *Fam82b* | 10503484 | NM_025476 | 4.36E-02 | -1.41 |
| *Atl2* | 10453062 | NM_019717 | 4.66E-02 | -1.41 |
| *Gm2451* | 10425903 | ENSMUST00000106238 | 2.95E-02 | -1.41 |
| *Mrpl38* | 10393125 | NM_024177 | 4.84E-02 | -1.39 |
| *---* | 10595046 | GENSCAN00000006758 | 3.98E-02 | -1.39 |
| *Tet1* | 10369690 | NM_027384 | 3.21E-02 | -1.39 |
| *Gapdh* | 10474239 | GU214026 | 2.94E-02 | -1.39 |
| *Gm6981* | 10593490 | NR_023357 | 3.59E-02 | -1.39 |
| *Gm5177* | 10361710 | NR_033630 | 2.46E-02 | -1.39 |
| *Abcc9* | 10549108 | NM_021041 | 4.25E-02 | -1.39 |
| *Wnk2* | 10409118 | NM_029361 | 3.10E-02 | -1.39 |
| *Pgp* | 10442454 | NM_025954 | 3.94E-02 | -1.39 |
| *Slc6a7* | 10459138 | NM_201353 | 3.09E-02 | -1.37 |
| *Eif4ebp1* | 10571162 | NM_007918 | 4.85E-02 | -1.37 |
| *Klhl4* | 10601519 | NM_172781 | 4.99E-02 | -1.37 |
| *Arhgap5* | 10395692 | NM_009706 | 4.79E-02 | -1.37 |
| *Gapdh* | 10368612 | BC092294 | 3.17E-02 | -1.37 |
| *Gapdh* | 10384493 | NM_008084 | 2.72E-02 | -1.37 |
| *Tceal3* | 10601874 | NM_001029978 | 4.93E-02 | -1.37 |
| *Gm10468* | 10395606 | ENSMUST00000101453 | 2.94E-02 | -1.37 |
| *Gm12070* | 10601567 | NR_002890 | 2.76E-02 | -1.37 |
| *Ssbp2* | 10406551 | NM_024272 | 2.47E-02 | -1.35 |
| *5730403B10Rik* | 10437443 | NM_025670 | 4.05E-02 | -1.35 |
| *Gm4609* | 10463064 | ENSMUST00000106443 | 2.95E-02 | -1.35 |
| *Parp6* | 10585905 | NM_029922 | 3.80E-02 | -1.35 |
| *Grsf1* | 10531133 | NM_178700 | 3.97E-02 | -1.35 |
| *Luzp1* | 10517463 | BC137786 | 4.90E-02 | -1.35 |
| *Gapdh* | 10466843 | BC092267 | 2.34E-02 | -1.33 |
| *Fgf20* | 10578281 | NM_030610 | 2.95E-02 | -1.33 |
| *3222401L13Rik* | 10455146 | ENSMUST00000097608 | 3.40E-02 | -1.32 |
| *Gapdh* | 10409200 | NM_008084 | 3.38E-02 | -1.32 |
| *Prdx6* | 10359422 | NM_007453 | 4.73E-02 | -1.32 |
| *Cd59a* | 10474229 | NM_001111060 | 3.80E-02 | -1.30 |
| *2610030H06Rik* | 10600031 | NM_001081356 | 3.89E-02 | -1.30 |
| *2810410L24Rik* | 10393749 | NR_030682 | 4.22E-02 | -1.30 |
| *Mrps18b* | 10450640 | NM_025878 | 1.88E-02 | -1.30 |
| *Pgcp* | 10423556 | NM_018755 | 4.79E-02 | -1.25 |
| *Dr1* | 10523955 | NM_026106 | 3.35E-02 | 1.23 |
| *Rab8a* | 10572757 | NM_023126 | 4.30E-02 | 1.23 |
| *Utp3* | 10522973 | NM_023054 | 3.35E-02 | 1.24 |
| *Klraq1* | 10447437 | NM_028658 | 4.65E-02 | 1.24 |
| *Ube2g2* | 10364293 | NM_019803 | 3.80E-02 | 1.25 |
| *Hdac1* | 10447004 | NM_008228 | 3.78E-02 | 1.25 |
| *Med15* | 10438198 | NM_033609 | 4.09E-02 | 1.25 |
| *Tyk2* | 10591446 | NM_018793 | 3.81E-02 | 1.26 |
| *Pi4k2a* | 10463211 | NM_145501 | 2.77E-02 | 1.26 |
| *Mmgt2* | 10376889 | NM_175002 | 4.66E-02 | 1.26 |
| *Dcaf7* | 10382010 | NM_027946 | 4.79E-02 | 1.26 |
| *Ncstn* | 10360205 | NM_021607 | 3.72E-02 | 1.26 |
| *Tbc1d22a* | 10426065 | NM_145476 | 3.51E-02 | 1.27 |
| *Hdac1* | 10516605 | NM_008228 | 3.09E-02 | 1.27 |
| *Pon2* | 10543004 | NM_183308 | 4.66E-02 | 1.27 |
| *---* | 10608661 | NM_198193.2 | 3.71E-02 | 1.27 |
| *Pfn1* | 10387932 | NM_011072 | 4.84E-02 | 1.28 |
| *Eps15* | 10506939 | NM_007943 | 4.66E-02 | 1.28 |
| *Dpp7* | 10480699 | NM_031843 | 3.39E-02 | 1.28 |
| *Tnfaip8* | 10455647 | NM_134131 | 4.71E-02 | 1.28 |
| *Nubp1* | 10433494 | NM_011955 | 3.30E-02 | 1.28 |
| *Pqlc2* | 10517689 | NM_145384 | 4.72E-02 | 1.28 |
| *Oat* | 10568568 | NM_016978 | 3.35E-02 | 1.29 |
| *Ripk1* | 10404506 | NM_009068 | 4.25E-02 | 1.29 |
| *Cdk9* | 10481670 | NM_130860 | 4.43E-02 | 1.29 |
| *Cyp4f16* | 10443869 | NM_024442 | 3.01E-02 | 1.30 |
| *Ran* | 10525983 | NM_009391 | 4.84E-02 | 1.30 |
| *Eif2ak3* | 10538939 | NM_010121 | 2.46E-02 | 1.30 |
| *Nup50* | 10425905 | NM_016714 | 4.57E-02 | 1.30 |
| *Fam38a* | 10582376 | NM_001037298 | 2.50E-02 | 1.30 |
| *E430025E21Rik* | 10428877 | NM_153548 | 2.93E-02 | 1.30 |
| *Heatr5a* | 10400254 | NM_177171 | 3.98E-02 | 1.31 |
| *Calm3* | 10560304 | NM_007590 | 3.37E-02 | 1.31 |
| *S100a10* | 10493995 | NM_009112 | 3.48E-02 | 1.31 |
| *Heatr7a* | 10424860 | NM_175457 | 4.05E-02 | 1.31 |
| *Rpl13a* | 10563101 | NM_009438 | 2.98E-02 | 1.31 |
| *Sh3gl1* | 10451907 | NM_013664 | 4.86E-02 | 1.31 |
| *Rgs19* | 10490736 | NM_026446 | 4.75E-02 | 1.31 |
| *Tbrg1* | 10592342 | NM_025289 | 4.10E-02 | 1.31 |
| *Plekhb2* | 10345423 | NM_145516 | 4.71E-02 | 1.32 |
| *Snx6* | 10400336 | NM_026998 | 4.58E-02 | 1.32 |
| *Prelid1* | 10405427 | NM_025596 | 5.00E-02 | 1.32 |
| *Nsf* | 10391963 | NM_008740 | 2.31E-02 | 1.32 |
| *Rrbp1* | 10488195 | NM_024281 | 3.66E-02 | 1.32 |
| *Diap1* | 10458430 | NM_007858 | 4.18E-02 | 1.32 |
| *Pxn* | 10524703 | NM_011223 | 4.74E-02 | 1.32 |
| *Vwa2* | 10464202 | NM_172840 | 3.20E-02 | 1.33 |
| *Sec11c* | 10456346 | NM_025468 | 4.66E-02 | 1.33 |
| *Clta* | 10504458 | NM_001080385 | 3.76E-02 | 1.33 |
| *Limk1* | 10534324 | NM_010717 | 2.76E-02 | 1.33 |
| *Eno2* | 10547807 | NM_013509 | 4.44E-02 | 1.33 |
| *Prkx* | 10605465 | NM_016979 | 3.01E-02 | 1.33 |
| *Calr* | 10580219 | NM_007591 | 3.64E-02 | 1.33 |
| *Myo9b* | 10572533 | NM_001142322 | 4.66E-02 | 1.33 |
| *Capn1* | 10465314 | NM_007600 | 1.85E-02 | 1.33 |
| *Hsd17b11* | 10531919 | NM_053262 | 3.46E-02 | 1.33 |
| *Tln1* | 10512514 | NM_011602 | 3.77E-02 | 1.34 |
| *Ifnar1* | 10436849 | NM_010508 | 3.63E-02 | 1.34 |
| *Dpp3* | 10464877 | NM_133803 | 3.69E-02 | 1.34 |
| *Tspan5* | 10496494 | NM_019571 | 4.45E-02 | 1.34 |
| *Mcl1* | 10494306 | NM_008562 | 4.91E-02 | 1.34 |
| *---* | 10344362 | --- | 4.25E-02 | 1.34 |
| *Gba* | 10493435 | NM_008094 | 3.03E-02 | 1.34 |
| *Tmem104* | 10382470 | NM_001033393 | 4.71E-02 | 1.34 |
| *D11Wsu99e* | 10392715 | NM_138598 | 2.46E-02 | 1.34 |
| *Myh9* | 10430201 | NM_022410 | 3.89E-02 | 1.34 |
| *Rab7l1* | 10349724 | NM_144875 | 2.94E-02 | 1.35 |
| *Dtx3l* | 10439268 | NM_001013371 | 4.66E-02 | 1.35 |
| *Ankrd33b* | 10427997 | NM_027496 | 3.80E-02 | 1.35 |
| *---* | 10341273 | --- | 3.80E-02 | 1.35 |
| *2310046K01Rik* | 10477052 | NM_001164819 | 4.83E-02 | 1.35 |
| *Pdk3* | 10605711 | NM_145630 | 2.85E-02 | 1.35 |
| *Dnase2a* | 10573461 | NM_010062 | 3.15E-02 | 1.35 |
| *E230008N13Rik* | 10504668 | NM_198660 | 4.70E-02 | 1.36 |
| *Man2a1* | 10446376 | NM_008549 | 2.70E-02 | 1.36 |
| *2610001J05Rik* | 10543226 | NR_024619 | 3.02E-02 | 1.36 |
| *Ext1* | 10428579 | NM_010162 | 3.21E-02 | 1.36 |
| *Zfp710* | 10554370 | NM_175433 | 2.95E-02 | 1.36 |
| *Mlkl* | 10581813 | NM_029005 | 3.69E-02 | 1.36 |
| *2310079N02Rik* | 10576561 | NM_025636 | 2.95E-02 | 1.36 |
| *Irak4* | 10426451 | NM_029926 | 3.24E-02 | 1.36 |
| *Vamp5* | 10545401 | NM_016872 | 2.50E-02 | 1.36 |
| *Crlf2* | 10532289 | NM_001164735 | 2.94E-02 | 1.36 |
| *Lrrfip1* | 10348493 | NM_008515 | 3.42E-02 | 1.37 |
| *Hk1* | 10369541 | NM_010438 | 4.25E-02 | 1.37 |
| *Heatr7a* | 10424894 | NM_175457 | 2.90E-02 | 1.37 |
| *Dock11* | 10599120 | NM_001009947 | 3.80E-02 | 1.37 |
| *Bin3* | 10416256 | NM_021328 | 2.78E-02 | 1.37 |
| *Rap1b* | 10372716 | NM_024457 | 4.10E-02 | 1.37 |
| *Pdia3* | 10475335 | NM_007952 | 3.50E-02 | 1.37 |
| *---* | 10608719 | NM_001008233.1 | 3.82E-02 | 1.37 |
| *Arhgef3* | 10413419 | NM_027871 | 3.19E-02 | 1.37 |
| *Cd47* | 10436182 | NM_010581 | 3.09E-02 | 1.37 |
| *Aldh1l2* | 10371332 | NM_153543 | 4.90E-02 | 1.37 |
| *Psma5* | 10365426 | NM_011967 | 3.55E-02 | 1.38 |
| *Ywhah* | 10521031 | NM_011738 | 2.46E-02 | 1.38 |
| *Dcakd* | 10391831 | NM_026551 | 4.80E-02 | 1.38 |
| *Arpc4* | 10540650 | NM_026552 | 2.35E-02 | 1.38 |
| *Srsf9* | 10524676 | NM_025573 | 4.75E-02 | 1.38 |
| *Cobl* | 10384423 | NM_172496 | 3.14E-02 | 1.38 |
| *Rnpep* | 10358038 | NM_145417 | 2.97E-02 | 1.38 |
| *Gng10* | 10505182 | NM_025277 | 4.27E-02 | 1.38 |
| *---* | 10343407 | --- | 4.05E-02 | 1.38 |
| *1110002B05Rik* | 10400321 | NM_134054 | 1.77E-02 | 1.38 |
| *Itpripl2* | 10567297 | NM_001033380 | 4.62E-02 | 1.38 |
| *Dbnl* | 10374068 | NM_001146308 | 2.46E-02 | 1.38 |
| *Arpc1b* | 10604763 | NM_023142 | 3.09E-02 | 1.38 |
| *Clcn5* | 10603289 | NM_016691 | 3.17E-02 | 1.38 |
| *Esd* | 10416411 | NM_016903 | 4.25E-02 | 1.38 |
| *Glb1* | 10589889 | NM_009752 | 2.46E-02 | 1.39 |
| *Nin* | 10400805 | NM_008697 | 4.90E-02 | 1.39 |
| *Itpripl2* | 10567299 | NM_001033380 | 2.21E-02 | 1.39 |
| *Mapk7* | 10386723 | NM_011841 | 3.43E-02 | 1.39 |
| *Klhl18* | 10597162 | NM_177771 | 1.83E-02 | 1.39 |
| *Slc15a4* | 10533993 | NM_133895 | 3.82E-02 | 1.39 |
| *Prkcz* | 10561335 | NM_008860 | 3.09E-02 | 1.39 |
| *Dnajb11* | 10434675 | NM_001190804 | 3.52E-02 | 1.39 |
| *Mvp* | 10568115 | NM_080638 | 4.84E-02 | 1.39 |
| *---* | 10343163 | --- | 3.27E-02 | 1.39 |
| *2610029G23Rik* | 10601335 | BC035042 | 4.24E-02 | 1.39 |
| *Pip5k1c* | 10365069 | NM_008844 | 2.95E-02 | 1.39 |
| *Iqgap2* | 10411235 | NM_027711 | 2.02E-02 | 1.39 |
| *Taok3* | 10524844 | NM_001081308 | 2.95E-02 | 1.39 |
| *Ap1b1* | 10373986 | NM_007454 | 1.97E-02 | 1.40 |
| *B4galt3* | 10351563 | NM_020579 | 3.09E-02 | 1.40 |
| *Wisp1* | 10424543 | NM_018865 | 2.95E-02 | 1.40 |
| *Sec11a* | 10565072 | NM_019951 | 1.47E-02 | 1.40 |
| *Snx30* | 10505224 | NM_172468 | 3.92E-02 | 1.40 |
| *Kcnk6* | 10561702 | NM_001033525 | 3.03E-02 | 1.40 |
| *---* | 10343704 | --- | 3.00E-02 | 1.40 |
| *Neu1* | 10444578 | NM_010893 | 3.48E-02 | 1.40 |
| *---* | 10340502 | --- | 4.22E-02 | 1.40 |
| *Nup43* | 10361651 | NM_145706 | 3.11E-02 | 1.40 |
| *Phc2* | 10508351 | NM_018774 | 2.95E-02 | 1.40 |
| *Esyt1* | 10373407 | NM_011843 | 3.80E-02 | 1.40 |
| *Crb3* | 10446224 | NM_177638 | 2.34E-02 | 1.40 |
| *Scamp2* | 10585721 | NM_022813 | 2.95E-02 | 1.40 |
| *Entpd7* | 10463282 | NM_053103 | 4.61E-02 | 1.40 |
| *Arid5a* | 10345445 | NM_001172205 | 3.09E-02 | 1.40 |
| *Sae1* | 10560260 | NM_019748 | 3.59E-02 | 1.41 |
| *Lasp1* | 10380823 | NM_010688 | 4.70E-02 | 1.41 |
| *Mgat2* | 10396074 | NM_146035 | 4.91E-02 | 1.41 |
| *P4hb* | 10393823 | NM_011032 | 2.98E-02 | 1.41 |
| *Nol10* | 10394749 | NM_001008421 | 4.40E-02 | 1.41 |
| *Kcnq1* | 10559276 | NM_008434 | 4.23E-02 | 1.41 |
| *Ptk2b* | 10420957 | NM_001162365 | 4.59E-02 | 1.41 |
| *Spryd3* | 10432923 | NM_001033277 | 2.60E-02 | 1.41 |
| *Slc39a1* | 10470948 | NM_013901 | 3.14E-02 | 1.41 |
| *Ctsz* | 10490212 | NM_022325 | 3.14E-02 | 1.41 |
| *Car9* | 10504337 | NM_139305 | 4.59E-02 | 1.41 |
| *---* | 10375485 | ENSMUST00000121448 | 2.95E-02 | 1.41 |
| *Atp6v1b2* | 10572146 | NM_007509 | 3.20E-02 | 1.41 |
| *Rab43* | 10546056 | NM_001039394 | 3.50E-02 | 1.42 |
| *Mafb* | 10489246 | NM_010658 | 4.91E-02 | 1.42 |
| *Wdr1* | 10529689 | NM_011715 | 2.93E-02 | 1.42 |
| *Nlrp1b* | 10388065 | NM_001162414 | 3.15E-02 | 1.42 |
| *Gmppb* | 10588836 | NM_177910 | 4.66E-02 | 1.42 |
| *Bak1* | 10449303 | NM_007523 | 3.22E-02 | 1.42 |
| *P2rx4* | 10525439 | NM_011026 | 1.47E-02 | 1.42 |
| *Cacnb3* | 10426611 | NM_007581 | 4.66E-02 | 1.42 |
| *Arpc1b* | 10527441 | NM_023142 | 2.34E-02 | 1.42 |
| *Lrrc16a* | 10408280 | NM_026825 | 3.98E-02 | 1.42 |
| *Lyl1* | 10573419 | NM_008535 | 4.49E-02 | 1.43 |
| *Cenpt* | 10581363 | NM_177150 | 2.89E-02 | 1.43 |
| *H2-M3* | 10445119 | NM_013819 | 2.96E-02 | 1.43 |
| *Tpcn2* | 10569656 | NM_146206 | 2.93E-02 | 1.43 |
| *Madd* | 10484941 | NM_001177721 | 2.38E-02 | 1.43 |
| *Me2* | 10459730 | NM_145494 | 1.83E-02 | 1.43 |
| *Ikbke* | 10357604 | NM_019777 | 4.75E-02 | 1.43 |
| *Casp1* | 10582985 | NM_009807 | 4.62E-02 | 1.43 |
| *---* | 10341595 | --- | 4.50E-02 | 1.43 |
| *Ppil5* | 10396068 | NM_001081406 | 4.75E-02 | 1.43 |
| *---* | 10341790 | --- | 3.39E-02 | 1.44 |
| *Rbm3* | 10603469 | NM_001166409 | 4.22E-02 | 1.44 |
| *5330426P16Rik* | 10439881 | NR_028300 | 3.63E-02 | 1.44 |
| *Erp29* | 10533316 | NM_026129 | 1.77E-02 | 1.44 |
| *Dennd1c* | 10452269 | NM_153551 | 4.54E-02 | 1.44 |
| *Pdia4* | 10544525 | NM_009787 | 2.60E-02 | 1.44 |
| *Nfkb2* | 10463599 | NM_019408 | 3.76E-02 | 1.44 |
| *---* | 10424437 | ENSMUST00000100608 | 1.62E-02 | 1.44 |
| *Lepre1* | 10507612 | NM_019783 | 2.93E-02 | 1.44 |
| *Ptpn1* | 10478897 | NM_011201 | 3.17E-02 | 1.44 |
| *Reep4* | 10416290 | NM_180588 | 4.84E-02 | 1.44 |
| *Prr13* | 10427235 | NM_025385 | 3.39E-02 | 1.44 |
| *---* | 10338228 | --- | 4.57E-02 | 1.45 |
| *Hspa5* | 10471586 | NM_022310 | 3.17E-02 | 1.45 |
| *Sbno2* | 10370721 | NM_183426 | 3.31E-02 | 1.45 |
| *Pkd2l1* | 10467962 | NM_181422 | 2.95E-02 | 1.45 |
| *D1Ertd622e* | 10356932 | NM_133825 | 4.79E-02 | 1.45 |
| *Prex1* | 10489784 | NM_177782 | 3.35E-02 | 1.45 |
| *Lrp1* | 10373223 | NM_008512 | 3.09E-02 | 1.45 |
| *Tpm3* | 10416940 | BC092045 | 3.09E-02 | 1.45 |
| *Kcnd1* | 10598448 | NM_008423 | 4.71E-02 | 1.45 |
| *Flna* | 10605256 | NM_010227 | 3.35E-02 | 1.45 |
| *Ahcy* | 10488816 | NM_016661 | 4.10E-02 | 1.45 |
| *Cercam* | 10470775 | NM_207298 | 2.87E-02 | 1.45 |
| *---* | 10342176 | --- | 4.37E-02 | 1.46 |
| *---* | 10368370 | GENSCAN00000041807 | 4.70E-02 | 1.46 |
| *Rbm3* | 10358454 | NM_001166409 | 3.69E-02 | 1.46 |
| *Snx2* | 10455738 | NM_026386 | 3.21E-02 | 1.46 |
| *Coro1c* | 10532753 | NM_011779 | 4.10E-02 | 1.46 |
| *2210404J11Rik* | 10441954 | AK019108 | 4.79E-02 | 1.46 |
| *S1pr2* | 10591412 | NM_010333 | 3.80E-02 | 1.46 |
| *Icam1* | 10583519 | NM_010493 | 3.09E-02 | 1.47 |
| *Naa38* | 10536595 | NM_133939 | 3.98E-02 | 1.47 |
| *Chmp4b* | 10477572 | NM_029362 | 2.34E-02 | 1.47 |
| *Ahcy* | 10344713 | NM_016661 | 3.24E-02 | 1.47 |
| *Asah1* | 10578361 | NM_019734 | 1.58E-02 | 1.47 |
| *Twf2* | 10588466 | NM_011876 | 1.88E-02 | 1.47 |
| *Rbm3* | 10556113 | NM_001166410 | 2.93E-02 | 1.47 |
| *Tnfrsf21* | 10445241 | NM_178589 | 2.96E-02 | 1.47 |
| *Pdia6* | 10394735 | NM_027959 | 1.77E-02 | 1.47 |
| *Nipal1* | 10522368 | NM_001081205 | 3.17E-02 | 1.47 |
| *BC034090* | 10358991 | AK129406 | 1.58E-02 | 1.47 |
| *1300002K09Rik* | 10504582 | NM_028788 | 4.99E-02 | 1.48 |
| *6430548M08Rik* | 10575993 | NM_172286 | 1.99E-02 | 1.48 |
| *Ap2m1* | 10434384 | NM_009679 | 1.97E-02 | 1.48 |
| *Cklf* | 10574456 | NM_001037841 | 2.95E-02 | 1.48 |
| *---* | 10436594 | AK076276 | 3.79E-02 | 1.48 |
| *Cdt1* | 10576140 | NM_026014 | 4.90E-02 | 1.48 |
| *Hsd17b2* | 10575833 | NM_008290 | 2.77E-02 | 1.48 |
| *Gins1* | 10476989 | NM_027014 | 1.61E-02 | 1.48 |
| *Ahcy* | 10439762 | NM_016661 | 3.37E-02 | 1.48 |
| *Prkcb* | 10557177 | NM_008855 | 3.11E-02 | 1.48 |
| *Slc13a3* | 10489705 | NM_054055 | 4.24E-02 | 1.48 |
| *Tubb2b // Tubb2b* | 10399419 | NM_023716 | 2.85E-02 | 1.48 |
| *Rab11fip5* | 10545827 | NM_001003955 | 4.59E-02 | 1.48 |
| *Itgb4* | 10382713 | NM_001005608 | 3.14E-02 | 1.48 |
| *Leprot* | 10506298 | NM_175036 | 1.87E-02 | 1.48 |
| *Adam17* | 10399605 | NM_009615 | 2.46E-02 | 1.48 |
| *Ckmt1* | 10475324 | NM_009897 | 4.40E-02 | 1.48 |
| *---* | 10339125 | --- | 3.81E-02 | 1.48 |
| *Snx5* | 10488237 | NM_024225 | 1.47E-02 | 1.48 |
| *2310004N24Rik* | 10382774 | NM_027107 | 1.28E-02 | 1.48 |
| *Def6* | 10443319 | NM_027185 | 2.14E-02 | 1.49 |
| *Fgd2* | 10443506 | NM_001159538 | 3.31E-02 | 1.49 |
| *Map3k8* | 10457225 | NM_007746 | 2.76E-02 | 1.49 |
| *Myo18b* | 10532590 | NM_028901 | 2.60E-02 | 1.49 |
| *Rnf19b* | 10508392 | NM_029219 | 1.58E-02 | 1.49 |
| *Dok1* | 10545623 | NM_010070 | 3.99E-02 | 1.49 |
| *Gas2l3* | 10371770 | NM_001033331 | 4.40E-02 | 1.49 |
| *Zmynd15* | 10377826 | NM_001029929 | 2.47E-02 | 1.49 |
| *Cdr2l* | 10382502 | NM_001080929 | 4.89E-02 | 1.49 |
| *Anxa9* | 10500140 | NM_001085383 | 1.47E-02 | 1.49 |
| *Birc3* | 10590801 | NM_007464 | 2.62E-02 | 1.49 |
| *Sfxn3* | 10463462 | NM_053197 | 3.98E-02 | 1.49 |
| *B4galnt1* | 10366848 | NM_008080 | 3.21E-02 | 1.49 |
| *Pycard* | 10568355 | NM_023258 | 2.65E-02 | 1.50 |
| *Slc39a1* | 10493709 | NM_013901 | 1.47E-02 | 1.50 |
| *Nfatc2* | 10489961 | NM_010899 | 2.95E-02 | 1.50 |
| *Dcaf15* | 10580085 | NM_172502 | 3.31E-02 | 1.50 |
| *Tbc1d9* | 10573128 | NM_001111304 | 4.56E-02 | 1.50 |
| *Fam167b* | 10516637 | NM_182783 | 2.95E-02 | 1.50 |
| *2310014H01Rik* | 10444932 | NM_001146711 | 4.75E-02 | 1.50 |
| *---* | 10343202 | --- | 3.74E-02 | 1.50 |
| *Iqgap1* | 10565018 | NM_016721 | 3.43E-02 | 1.50 |
| *Pip4k2a* | 10480347 | NM_008845 | 2.73E-02 | 1.50 |
| *Cd33* | 10562709 | NM_001111058 | 4.17E-02 | 1.50 |
| *Srd5a3* | 10522589 | NM_020611 | 2.83E-02 | 1.50 |
| *Nrm* | 10450611 | NM_134122 | 2.77E-02 | 1.50 |
| *---* | 10342233 | --- | 2.88E-02 | 1.51 |
| *1190002A17Rik* | 10470614 | NM_001033874 | 4.75E-02 | 1.51 |
| *---* | 10342490 | --- | 2.62E-02 | 1.51 |
| *Nasp* | 10515337 | NM_016777 | 2.99E-02 | 1.51 |
| *Tnfrsf11a* | 10349051 | NM_009399 | 2.36E-02 | 1.51 |
| *Hexa* | 10585874 | NM_010421 | 2.47E-02 | 1.51 |
| *Arrb2* | 10377804 | NM_145429 | 2.02E-02 | 1.51 |
| *Slc2a1* | 10507594 | NM_011400 | 4.76E-02 | 1.51 |
| *---* | 10342764 | --- | 3.14E-02 | 1.51 |
| *---* | 10338657 | --- | 4.75E-02 | 1.51 |
| *Arpc3* | 10525397 | NM_019824 | 1.84E-02 | 1.51 |
| *Scamp5* | 10593927 | NM_020270 | 2.48E-02 | 1.51 |
| *Pgm1* | 10522009 | NM_025700 | 2.97E-02 | 1.51 |
| *Gria3* | 10599348 | NM_016886 | 4.22E-02 | 1.51 |
| *2210404J11Rik* | 10441933 | NM_001039552 | 2.97E-02 | 1.51 |
| *Man1c1* | 10517287 | NM_207237 | 1.88E-02 | 1.52 |
| *Ly6g5b* | 10450431 | NM_148939 | 3.84E-02 | 1.52 |
| *Zdhhc23* | 10439542 | NM_001007460 | 3.98E-02 | 1.52 |
| *C1qc* | 10517513 | NM_007574 | 4.68E-02 | 1.52 |
| *Lrrc8e* | 10569923 | NM_028175 | 2.18E-02 | 1.52 |
| *Mctp1* | 10406334 | NM_030174 | 2.23E-02 | 1.52 |
| *Tspan32* | 10559248 | NM_020286 | 3.77E-02 | 1.52 |
| *Anxa4* | 10545958 | NM_013471 | 1.28E-02 | 1.52 |
| *Nt5c* | 10392936 | NM_015807 | 2.36E-02 | 1.53 |
| *Cerk* | 10431266 | NM_145475 | 2.76E-02 | 1.53 |
| *Slfn10-ps* | 10389151 | NM_181542 | 4.85E-02 | 1.53 |
| *Atp7a* | 10601360 | NM_001109757 | 1.83E-02 | 1.53 |
| *Manba* | 10496302 | NM_027288 | 1.59E-02 | 1.53 |
| *Panx1* | 10590983 | NM_019482 | 3.93E-02 | 1.53 |
| *Best1* | 10465881 | NM_011913 | 4.50E-02 | 1.53 |
| *Myo1e* | 10586781 | NM_181072 | 2.44E-02 | 1.53 |
| *Plec* | 10429802 | NM_201391 | 1.29E-02 | 1.53 |
| *Gsg2* | 10388234 | NM_010353 | 4.66E-02 | 1.53 |
| *Ppp2r5c* | 10398455 | NM_001135001 | 4.77E-02 | 1.53 |
| *Slc9a3r1* | 10382462 | NM_012030 | 3.69E-02 | 1.53 |
| *Plekha2* | 10577792 | NM_031257 | 3.22E-02 | 1.53 |
| *Ninj1* | 10404996 | NM_013610 | 2.95E-02 | 1.54 |
| *Fam49b* | 10428983 | NM_144846 | 2.54E-02 | 1.54 |
| *Plcl2* | 10445867 | NM_013880 | 2.39E-02 | 1.54 |
| *Ucp2* | 10555389 | NM_011671 | 3.31E-02 | 1.54 |
| *Rcn1* | 10485645 | NM_009037 | 3.69E-02 | 1.54 |
| *Ifngr2* | 10436865 | NM_008338 | 3.09E-02 | 1.54 |
| *Uap1l1* | 10480714 | NM_001033293 | 1.99E-02 | 1.54 |
| *Pik3cd* | 10518686 | NM_001164049 | 4.53E-02 | 1.54 |
| *---* | 10343028 | --- | 2.33E-02 | 1.54 |
| *Tpm4* | 10448202 | NM_001001491 | 3.80E-02 | 1.54 |
| *Hmga1* | 10383479 | NM_016660 | 1.62E-02 | 1.54 |
| *---* | 10425046 | --- | 2.95E-02 | 1.55 |
| *Sh3pxd2b* | 10375065 | NM_177364 | 3.09E-02 | 1.55 |
| *Fen1* | 10465912 | NM_007999 | 4.10E-02 | 1.55 |
| *Hnrnpa3* | 10503370 | NM_146130 | 3.01E-02 | 1.55 |
| *Slamf9* | 10351792 | NM_029612 | 1.46E-02 | 1.55 |
| *Ccdc109b* | 10502156 | NM_025779 | 4.66E-02 | 1.55 |
| *Tmsb10* | 10545479 | NM_025284 | 2.94E-02 | 1.55 |
| *---* | 10338708 | --- | 3.64E-02 | 1.55 |
| *Tmsb10* | 10551009 | NM_025284 | 3.21E-02 | 1.55 |
| *Slc38a1* | 10431872 | NM_134086 | 3.22E-02 | 1.55 |
| *Sptlc2* | 10401781 | NM_011479 | 1.86E-02 | 1.55 |
| *Hnrnpa3* | 10473008 | NM_146130 | 3.58E-02 | 1.56 |
| *Phlda1* | 10366346 | NM_009344 | 1.23E-02 | 1.56 |
| *Nup93* | 10574033 | NM_172410 | 1.58E-02 | 1.56 |
| *Sh3bgrl3* | 10517169 | NM_080559 | 3.21E-02 | 1.56 |
| *Ckb* | 10402708 | NM_021273 | 2.95E-02 | 1.56 |
| *Lrrc33* | 10439058 | NM_146069 | 2.96E-02 | 1.56 |
| *Trem2* | 10445781 | NM_031254 | 4.89E-02 | 1.56 |
| *Sash3* | 10599487 | NM_028773 | 2.33E-02 | 1.57 |
| *---* | 10338159 | --- | 4.44E-02 | 1.57 |
| *Gna15* | 10371220 | NM_010304 | 1.39E-02 | 1.57 |
| *Cmtm7* | 10597461 | NM_133978 | 3.17E-02 | 1.57 |
| *B3gnt3* | 10579602 | NM_028189 | 2.78E-02 | 1.57 |
| *Gm22* | 10576088 | AK122564 | 3.03E-02 | 1.57 |
| *Eme1* | 10390050 | NM_177752 | 2.70E-02 | 1.57 |
| *Rel* | 10384725 | NM_009044 | 4.33E-02 | 1.57 |
| *Atp6ap2* | 10598664 | NM_027439 | 2.48E-02 | 1.57 |
| *Abhd2* | 10554269 | NM_018811 | 2.46E-02 | 1.57 |
| *Serinc5* | 10406598 | NM_172588 | 3.85E-02 | 1.57 |
| *Tbc1d14* | 10529549 | NM_001113362 | 1.99E-02 | 1.57 |
| *Rasal1* | 10525134 | NM_013832 | 3.09E-02 | 1.57 |
| *Msn* | 10600836 | NM_010833 | 1.39E-02 | 1.58 |
| *Incenp* | 10465861 | NM_016692 | 4.56E-02 | 1.58 |
| *Ceacam2* | 10561055 | NM_001113368 | 2.95E-02 | 1.58 |
| *Dnmt1* | 10591369 | NM_010066 | 2.70E-02 | 1.58 |
| *Spns2* | 10388194 | NM_153060 | 2.47E-02 | 1.58 |
| *Agtrap* | 10518455 | NM_009642 | 1.85E-02 | 1.58 |
| *Plxnb2* | 10431424 | NM_138749 | 2.90E-02 | 1.58 |
| *Slc2a6* | 10481164 | NM_172659 | 3.72E-02 | 1.59 |
| *Fzd1* | 10527936 | NM_021457 | 2.87E-02 | 1.59 |
| *Vill* | 10590073 | NM_001164567 | 1.31E-02 | 1.59 |
| *Limd2* | 10392063 | NM_172397 | 3.39E-02 | 1.59 |
| *Frrs1* | 10495596 | NM_001113478 | 4.86E-02 | 1.59 |
| *Emilin1* | 10520604 | NM_133918 | 3.34E-02 | 1.59 |
| *AI467606* | 10557571 | NM_178901 | 2.19E-02 | 1.59 |
| *Tgfb1* | 10551185 | NM_011577 | 2.35E-02 | 1.59 |
| *Cndp2* | 10460085 | NM_023149 | 1.28E-02 | 1.59 |
| *Colec12* | 10453747 | NM_130449 | 2.03E-02 | 1.59 |
| *Apob48r* | 10557434 | NM_138310 | 2.73E-02 | 1.59 |
| *---* | 10338486 | --- | 3.17E-02 | 1.60 |
| *Cfp* | 10603860 | NM_008823 | 4.10E-02 | 1.60 |
| *Myd88* | 10597648 | NM_010851 | 2.85E-02 | 1.60 |
| *Tnfsf13b* | 10570018 | NM_033622 | 2.33E-02 | 1.60 |
| *Cnr2* | 10509122 | NM_009924 | 2.95E-02 | 1.60 |
| *Lpcat1* | 10406031 | NM_145376 | 3.31E-02 | 1.60 |
| *Lcp2* | 10375145 | NM_010696 | 2.23E-02 | 1.60 |
| *Plxnc1* | 10372028 | NM_018797 | 4.79E-02 | 1.61 |
| *Slc38a6* | 10396383 | NM_001037717 | 2.02E-02 | 1.61 |
| *Pla2g15* | 10574976 | NM_133792 | 1.28E-02 | 1.61 |
| *Brip1* | 10389395 | NM_178309 | 3.69E-02 | 1.61 |
| *Rab31* | 10452485 | NM_133685 | 3.35E-02 | 1.61 |
| *Ostc* | 10502191 | NM_025509 | 2.76E-02 | 1.61 |
| *Csf2rb* | 10425066 | NM_007780 | 2.35E-02 | 1.62 |
| *Ada* | 10489391 | NM_007398 | 2.36E-02 | 1.62 |
| *Arl11* | 10415778 | NM_177337 | 4.40E-02 | 1.62 |
| *Aldh3b1* | 10464560 | NM_026316 | 1.61E-02 | 1.62 |
| *Hnrnpa3* | 10600593 | NM_053263 | 2.77E-02 | 1.62 |
| *Plekho1* | 10500295 | NM_023320 | 2.33E-02 | 1.63 |
| *Batf* | 10397359 | NM_016767 | 3.46E-02 | 1.63 |
| *Hn1l* | 10448803 | NM_198937 | 2.85E-02 | 1.63 |
| *Man2b1* | 10573583 | NM_010764 | 3.63E-02 | 1.63 |
| *Il17ra* | 10541246 | NM_008359 | 4.71E-02 | 1.63 |
| *Il2rg* | 10606016 | NM_013563 | 2.50E-02 | 1.63 |
| *Rab3il1* | 10461408 | NM_144538 | 4.75E-02 | 1.63 |
| *Cdhr4* | 10588819 | NM_001122635 | 3.71E-02 | 1.63 |
| *Tagln2* | 10351825 | NM_178598 | 4.18E-02 | 1.63 |
| *Tcirg1* | 10464529 | NM_016921 | 2.97E-02 | 1.63 |
| *---* | 10340638 | --- | 3.77E-02 | 1.63 |
| *Cit* | 10524790 | NM_007708 | 4.09E-02 | 1.63 |
| *Runx3* | 10509030 | NM_019732 | 4.89E-02 | 1.64 |
| *Hnrnpa3* | 10599972 | ENSMUST00000090792 | 3.09E-02 | 1.64 |
| *Wdfy4* | 10418848 | NM_001146022 | 3.81E-02 | 1.64 |
| *Kif14* | 10350297 | NM_001081258 | 3.14E-02 | 1.64 |
| *Ehd4* | 10486396 | NM_133838 | 2.99E-02 | 1.64 |
| *Slc25a43* | 10599207 | NM_001085497 | 2.93E-02 | 1.64 |
| *Wdfy4* | 10418868 | NM_001146022 | 2.98E-02 | 1.64 |
| *Chaf1b* | 10437040 | NM_028083 | 4.40E-02 | 1.64 |
| *Nt5dc2* | 10413710 | NM_027289 | 3.83E-02 | 1.64 |
| *Pik3cg* | 10399924 | NM_020272 | 1.58E-02 | 1.64 |
| *Hnrnpa3* | 10528200 | NM_053263 | 2.46E-02 | 1.65 |
| *Sap30* | 10578763 | NM_021788 | 3.63E-02 | 1.65 |
| *Pqlc3* | 10399540 | NM_172574 | 2.03E-02 | 1.65 |
| *Sdf2l1* | 10438098 | NM_022324 | 3.80E-02 | 1.65 |
| *5031439G07Rik* | 10431170 | NM_001033273 | 1.28E-02 | 1.65 |
| *Lypd3* | 10550980 | NM_133743 | 4.25E-02 | 1.65 |
| *Bin2* | 10432640 | ENSMUST00000100198 | 4.91E-02 | 1.65 |
| *---* | 10342973 | --- | 4.77E-02 | 1.65 |
| *Litaf* | 10437687 | NM_019980 | 3.80E-02 | 1.65 |
| *Fam129b* | 10471535 | NM_146119 | 3.64E-02 | 1.65 |
| *Card9* | 10480956 | NM_001037747 | 1.46E-02 | 1.65 |
| *Laptm5* | 10508663 | NM_010686 | 2.77E-02 | 1.66 |
| *Bid* | 10547531 | NM_007544 | 1.39E-02 | 1.66 |
| *Ifi30* | 10579347 | NM_023065 | 3.92E-02 | 1.66 |
| *Fam110c* | 10395155 | NM_027828 | 3.37E-02 | 1.66 |
| *Grn* | 10381588 | NM_008175 | 1.58E-02 | 1.66 |
| *Naip2* | 10411595 | NM_010872 | 1.77E-02 | 1.66 |
| *Kmo* | 10352000 | NM_133809 | 2.33E-02 | 1.66 |
| *Fam105a* | 10427918 | BC052328 | 3.19E-02 | 1.66 |
| *Klra2* | 10548552 | NM_001170851 | 3.76E-02 | 1.66 |
| *Ptpn7* | 10350102 | NM_177081 | 1.83E-02 | 1.66 |
| *Hnrnpa3* | 10515113 | NM_053263 | 2.46E-02 | 1.67 |
| *Hnrnpa3* | 10344741 | NM_053263 | 2.46E-02 | 1.67 |
| *---* | 10519713 | GENSCAN00000007344 | 1.41E-02 | 1.67 |
| *---* | 10343336 | --- | 3.81E-02 | 1.67 |
| *Tnfaip8l2* | 10500100 | NM_027206 | 1.26E-02 | 1.67 |
| *Fam111a* | 10461723 | BC038020 | 1.85E-02 | 1.67 |
| *Cln6* | 10586110 | NM_001033175 | 1.31E-02 | 1.67 |
| *Plau* | 10413047 | NM_008873 | 3.09E-02 | 1.67 |
| *Cpt1a* | 10460157 | NM_013495 | 1.99E-02 | 1.67 |
| *Arhgap30* | 10351603 | NM_001005508 | 2.50E-02 | 1.67 |
| *Hnrnpa3* | 10437222 | NM_053263 | 2.50E-02 | 1.67 |
| *Stard3nl* | 10407946 | NM_024270 | 1.76E-02 | 1.67 |
| *Tmem173* | 10458314 | NM_028261 | 3.31E-02 | 1.67 |
| *Cd48* | 10351658 | NM_007649 | 2.33E-02 | 1.68 |
| *Pstpip1* | 10585555 | NM_011193 | 2.65E-02 | 1.68 |
| *Rasgef1a* | 10541114 | NM_027526 | 2.00E-02 | 1.68 |
| *Cdh1* | 10575052 | NM_009864 | 4.66E-02 | 1.69 |
| *Cd86* | 10439312 | NM_019388 | 3.10E-02 | 1.69 |
| *Ptplad2* | 10514275 | NM_025760 | 3.17E-02 | 1.69 |
| *AU021092* | 10437552 | NM_001033220 | 2.98E-02 | 1.69 |
| *Gcnt1* | 10466521 | NM_173442 | 1.28E-02 | 1.69 |
| *Hnrnpa3* | 10492045 | NM_053263 | 2.92E-02 | 1.69 |
| *Prr15* | 10538373 | NM_030024 | 1.28E-02 | 1.69 |
| *Arhgdib* | 10548892 | NM_007486 | 4.43E-02 | 1.69 |
| *C1qa* | 10517517 | NM_007572 | 2.46E-02 | 1.69 |
| *Dna2* | 10363575 | NM_177372 | 2.93E-02 | 1.69 |
| *Gpr35* | 10348645 | NM_022320 | 2.47E-02 | 1.69 |
| *Cd80* | 10435704 | NM_009855 | 1.28E-02 | 1.69 |
| *Sykb* | 10405216 | NM_011518 | 1.39E-02 | 1.69 |
| *---* | 10344502 | --- | 2.02E-02 | 1.69 |
| *Efemp2* | 10460603 | NM_021474 | 3.09E-02 | 1.70 |
| *Sla* | 10429128 | NM_001029841 | 3.78E-02 | 1.70 |
| *Arhgap11a* | 10485963 | NM_181416 | 3.50E-02 | 1.70 |
| *Ubash3b* | 10592515 | NM_176860 | 2.23E-02 | 1.70 |
| *Pik3r5* | 10377265 | NM_177320 | 1.83E-02 | 1.70 |
| *Trip13* | 10410560 | NM_027182 | 3.29E-02 | 1.70 |
| *Lhfpl2* | 10406676 | NM_172589 | 3.23E-02 | 1.70 |
| *Efemp1* | 10374777 | NM_146015 | 4.74E-02 | 1.70 |
| *Cfh* | 10358339 | NM_009888 | 4.70E-02 | 1.71 |
| *Plk5* | 10364792 | NM_183152 | 3.29E-02 | 1.71 |
| *Lilrb4* | 10363082 | NM_013532 | 3.74E-02 | 1.71 |
| *Sh3bp2* | 10521205 | NM_001145859 | 2.96E-02 | 1.71 |
| *BC021614* | 10464594 | BC034269 | 1.23E-02 | 1.71 |
| *Tfpi2* | 10542953 | NM_009364 | 3.09E-02 | 1.71 |
| *Ttk* | 10587508 | NM_009445 | 3.49E-02 | 1.71 |
| *Clec4a1* | 10541555 | NM_199311 | 3.17E-02 | 1.71 |
| *Hk3* | 10409376 | NM_001033245 | 1.62E-02 | 1.72 |
| *Nans* | 10504743 | NM_053179 | 1.47E-02 | 1.72 |
| *Lrp8* | 10506714 | NR_033496 | 1.69E-02 | 1.72 |
| *---* | 10344453 | --- | 3.80E-02 | 1.72 |
| *Selplg* | 10532744 | NM_009151 | 3.11E-02 | 1.72 |
| *Bcl3* | 10560685 | NM_033601 | 2.46E-02 | 1.72 |
| *Bex6* | 10435009 | NM_001033539 | 3.46E-02 | 1.72 |
| *Ccnf* | 10448506 | NM_007634 | 3.63E-02 | 1.72 |
| *Arhgap9* | 10366886 | NM_146011 | 1.30E-02 | 1.73 |
| *Tifab* | 10409567 | NM_001168615 | 1.42E-02 | 1.73 |
| *Cd300e* | 10392839 | NM_172050 | 1.23E-02 | 1.73 |
| *Ptpn6* | 10547769 | NM_013545 | 1.47E-02 | 1.73 |
| *Psd4* | 10469828 | NM_177611 | 1.39E-02 | 1.73 |
| *Sema4d* | 10409240 | NM_013660 | 3.14E-02 | 1.73 |
| *Gpr137b-ps* | 10407792 | NR_003568 | 2.33E-02 | 1.74 |
| *Arhgap4* | 10605143 | NM_138630 | 1.39E-02 | 1.74 |
| *Brca1* | 10391461 | NM_009764 | 3.58E-02 | 1.74 |
| *Asf1b* | 10573261 | NM_024184 | 3.14E-02 | 1.74 |
| *Mcm10* | 10479811 | NM_027290 | 4.37E-02 | 1.74 |
| *Rell1* | 10530130 | NM_145923 | 1.83E-02 | 1.74 |
| *Ckap4* | 10371387 | NM_175451 | 4.25E-02 | 1.74 |
| *---* | 10338272 | --- | 2.46E-02 | 1.74 |
| *Cyth4* | 10425092 | NM_028195 | 2.67E-02 | 1.74 |
| *2210404J11Rik* | 10441952 | NM_001039552 | 3.21E-02 | 1.74 |
| *Plvap* | 10579525 | NM_032398 | 2.83E-02 | 1.75 |
| *Serpinb8* | 10349174 | NM_011459 | 2.96E-02 | 1.75 |
| *Plekho2* | 10594540 | NM_153119 | 1.46E-02 | 1.75 |
| *Sbsn* | 10552037 | NM_172205 | 1.41E-02 | 1.75 |
| *Espl1* | 10427166 | NM_001014976 | 1.86E-02 | 1.75 |
| *Nfkbie* | 10445412 | NM_008690 | 3.03E-02 | 1.75 |
| *P2ry12* | 10498371 | NM_027571 | 1.52E-02 | 1.75 |
| *Creld2* | 10426098 | NM_029720 | 2.00E-02 | 1.75 |
| *Dock2* | 10385118 | NM_033374 | 1.88E-02 | 1.75 |
| *Bard1* | 10355329 | NM_007525 | 3.35E-02 | 1.76 |
| *Tnfrsf1b* | 10518300 | NM_011610 | 1.59E-02 | 1.76 |
| *D10Bwg1379e* | 10368101 | NM_001033258 | 3.03E-02 | 1.76 |
| *Slco2a1* | 10588263 | NM_033314 | 3.10E-02 | 1.76 |
| *Col3a1* | 10346015 | NM_009930 | 4.25E-02 | 1.76 |
| *Arhgap25* | 10546010 | NM_001037727 | 1.47E-02 | 1.76 |
| *Trpv2* | 10376868 | NM_011706 | 3.14E-02 | 1.76 |
| *Gusb* | 10534102 | NM_010368 | 1.38E-02 | 1.76 |
| *Sirpa* | 10476021 | NM_007547 | 1.99E-02 | 1.77 |
| *Susd3* | 10409162 | NM_025491 | 2.93E-02 | 1.77 |
| *Irf5* | 10536898 | NM_012057 | 2.33E-02 | 1.77 |
| *Hmha1* | 10364650 | NM_001142701 | 2.65E-02 | 1.77 |
| *Rgs10* | 10568392 | NM_026418 | 1.88E-02 | 1.77 |
| *Lass6* | 10472501 | NM_172856 | 1.39E-02 | 1.77 |
| *Efhd2* | 10518069 | NM_025994 | 2.48E-02 | 1.77 |
| *Cybb* | 10603551 | NM_007807 | 2.58E-02 | 1.77 |
| *5730559C18Rik* | 10358177 | BC053100 | 3.48E-02 | 1.77 |
| *Cstb* | 10364375 | NM_007793 | 3.57E-02 | 1.78 |
| *Cd84* | 10351679 | NM_013489 | 2.75E-02 | 1.78 |
| *Rad54l* | 10515257 | NM_009015 | 3.21E-02 | 1.78 |
| *Samsn1* | 10440393 | NM_023380 | 4.33E-02 | 1.78 |
| *Lbh* | 10446763 | NM_029999 | 1.23E-02 | 1.78 |
| *Slamf8* | 10360306 | NM_029084 | 3.63E-02 | 1.78 |
| *---* | 10341968 | --- | 2.78E-02 | 1.79 |
| *Pld4* | 10398907 | NM_178911 | 3.54E-02 | 1.79 |
| *BC006965* | 10392687 | NR_024085 | 3.78E-02 | 1.79 |
| *Slfn2* | 10379630 | NM_011408 | 2.35E-02 | 1.79 |
| *Cldn3* | 10526241 | NM_009902 | 4.50E-02 | 1.79 |
| *A530040E14Rik* | 10347921 | BC100303 | 2.34E-02 | 1.79 |
| *Plcg2* | 10575799 | NM_172285 | 1.28E-02 | 1.79 |
| *Clic1* | 10444658 | NM_033444 | 1.58E-02 | 1.79 |
| *Cxcl16* | 10387890 | NM_023158 | 2.34E-02 | 1.79 |
| *Tpbg* | 10587554 | NM_011627 | 2.88E-02 | 1.79 |
| *Cysltr1* | 10606355 | NM_021476 | 1.90E-02 | 1.80 |
| *Tubb2b* | 10408613 | NM_023716 | 2.34E-02 | 1.80 |
| *Fyb* | 10422760 | NM_011815 | 1.23E-02 | 1.80 |
| *Fam84a* | 10399465 | NM_029007 | 3.31E-02 | 1.80 |
| *BC006965* | 10392685 | NR_024085 | 2.70E-02 | 1.80 |
| *Myo7a* | 10565634 | NM_008663 | 1.59E-02 | 1.80 |
| *P2ry6* | 10565958 | NM_183168 | 3.37E-02 | 1.80 |
| *Tmem178* | 10447120 | NM_026516 | 1.83E-02 | 1.80 |
| *---* | 10461636 | ENSMUST00000087884 | 4.49E-02 | 1.80 |
| *Tmem82* | 10517980 | NM_145987 | 3.35E-02 | 1.80 |
| *Slc16a3* | 10383502 | NM_001038653 | 1.87E-02 | 1.81 |
| *Ticam2* | 10458816 | NM_173394 | 1.15E-02 | 1.81 |
| *Hcls1* | 10435565 | NM_008225 | 1.83E-02 | 1.81 |
| *---* | 10343995 | --- | 3.48E-02 | 1.81 |
| *1700025G04Rik* | 10358717 | NM_197990 | 4.66E-02 | 1.81 |
| *Ptprc* | 10358224 | NM_001111316 | 3.51E-02 | 1.81 |
| *Fam20a* | 10392464 | NM_153782 | 3.95E-02 | 1.81 |
| *Rasgrp4* | 10551696 | NM_001174155 | 3.17E-02 | 1.82 |
| *Inpp5d* | 10348244 | NM_010566 | 2.47E-02 | 1.82 |
| *Syn2* | 10540880 | NM_001111015 | 3.40E-02 | 1.82 |
| *F630028O10Rik* | 10600852 | NR_030718 | 1.23E-02 | 1.82 |
| *Fblim1* | 10517967 | NM_133754 | 3.78E-02 | 1.83 |
| *Csf2rb2* | 10430302 | NM_007781 | 3.98E-02 | 1.83 |
| *Stab1* | 10418506 | NM_138672 | 1.47E-02 | 1.83 |
| *Atp8b4* | 10487208 | NM_001080944 | 1.21E-02 | 1.83 |
| *Cd300lb* | 10392796 | NM_199221 | 2.35E-02 | 1.83 |
| *Sgol2* | 10346365 | NM_199007 | 2.34E-02 | 1.83 |
| *Flt3* | 10535780 | NM_010229 | 3.63E-02 | 1.84 |
| *Cdca2* | 10421029 | NM_175384 | 4.75E-02 | 1.84 |
| *Krt8* | 10432918 | NM_031170 | 3.76E-02 | 1.84 |
| *Adcy7* | 10573747 | NM_007406 | 1.88E-02 | 1.84 |
| *Evi2a* | 10388958 | NM_001033711 | 2.23E-02 | 1.84 |
| *Svep1* | 10513208 | NM_022814 | 4.40E-02 | 1.84 |
| *Lgmn* | 10402268 | NM_011175 | 1.47E-02 | 1.85 |
| *Atf3* | 10361091 | NM_007498 | 1.69E-02 | 1.85 |
| *Pik3ap1* | 10467578 | NM_031376 | 1.39E-02 | 1.86 |
| *Rac2* | 10430372 | NM_009008 | 4.02E-02 | 1.86 |
| *Ap1s2* | 10603051 | NM_026887 | 2.35E-02 | 1.86 |
| *Gmfg* | 10542981 | NM_022024 | 1.28E-02 | 1.86 |
| *2010002N04Rik* | 10459071 | NM_134133 | 3.98E-02 | 1.87 |
| *Ctsc* | 10554789 | NM_009982 | 2.00E-02 | 1.87 |
| *Gjb2* | 10415604 | NM_008125 | 2.95E-02 | 1.87 |
| *Clec4a3* | 10541564 | NM_153197 | 1.83E-02 | 1.87 |
| *I830127L07Rik* | 10429580 | ENSMUST00000100541 | 2.34E-02 | 1.88 |
| *Mxd3* | 10409424 | NM_016662 | 2.98E-02 | 1.88 |
| *---* | 10608655 | NM_010400.1 | 2.03E-02 | 1.88 |
| *---* | 10599213 | ENSMUST00000082596 | 3.69E-02 | 1.88 |
| *Tnfrsf23* | 10569504 | NM_024290 | 4.30E-02 | 1.88 |
| *Gp49a* | 10363070 | NM_008147 | 3.76E-02 | 1.88 |
| *Lpxn* | 10461765 | NM_134152 | 2.58E-02 | 1.88 |
| *Cd109* | 10587383 | NM_153098 | 1.88E-02 | 1.88 |
| *---* | 10339136 | --- | 2.70E-02 | 1.88 |
| *Card14* | 10383109 | NM_130886 | 1.99E-02 | 1.89 |
| *Galnt6* | 10432661 | NM_001161767 | 2.34E-02 | 1.89 |
| *A4galt* | 10430968 | NM_001170954 | 1.28E-02 | 1.90 |
| *Emr1* | 10446282 | NM_010130 | 1.58E-02 | 1.90 |
| *Klrb1a* | 10548286 | NM_010737 | 2.46E-02 | 1.90 |
| *Aoah* | 10403871 | NM_012054 | 1.58E-02 | 1.90 |
| *Cd200r1 // Cd200r1* | 10435907 | NM_021325 | 1.58E-02 | 1.90 |
| *Ncf1* | 10534202 | NM_010876 | 1.41E-02 | 1.90 |
| *Olfml3* | 10500808 | NM_133859 | 1.23E-02 | 1.90 |
| *Cfb* | 10450325 | NM_008198 | 3.56E-03 | 1.91 |
| *Lat2* | 10534303 | NM_020044 | 2.97E-02 | 1.91 |
| *Emp1* | 10542355 | NM_010128 | 2.34E-02 | 1.91 |
| *---* | 10392822 | ENSMUST00000106578 | 1.46E-02 | 1.91 |
| *Hck* | 10477250 | NM_010407 | 2.34E-02 | 1.91 |
| *Tgfbi* | 10405587 | NM_009369 | 3.63E-02 | 1.91 |
| *Myo18b* | 10532584 | NM_028901 | 2.30E-02 | 1.91 |
| *Mefv* | 10437243 | NM_001161790 | 2.33E-02 | 1.91 |
| *Dhrs9* | 10472538 | NM_175512 | 3.11E-02 | 1.91 |
| *Kcnab2* | 10518927 | NM_010598 | 1.28E-02 | 1.91 |
| *Kcnn4* | 10550877 | NM_008433 | 1.99E-02 | 1.91 |
| *Fcgr3* | 10360040 | NM_010188 | 3.37E-02 | 1.91 |
| *Ly86* | 10404606 | NM_010745 | 2.96E-02 | 1.92 |
| *Cdc20* | 10515744 | NM_023223 | 2.95E-02 | 1.92 |
| *Vav1* | 10446253 | NM_011691 | 1.69E-02 | 1.92 |
| *Sel1l3* | 10530059 | NM_172710 | 2.46E-02 | 1.92 |
| *Lilra6* | 10559478 | NM_011090 | 4.86E-03 | 1.92 |
| *Fam189a2* | 10466735 | NM_001114174 | 4.84E-02 | 1.92 |
| *Rnf183* | 10513587 | NM_153504 | 8.59E-03 | 1.92 |
| *Ncf2* | 10412123 | NM_010877 | 2.95E-02 | 1.92 |
| *Renbp* | 10605181 | NM_023132 | 2.95E-02 | 1.92 |
| *Cyp4f18* | 10579636 | NM_024444 | 1.58E-02 | 1.92 |
| *Il21r* | 10557342 | NM_021887 | 4.70E-02 | 1.93 |
| *Fermt3* | 10465587 | NM_153795 | 1.39E-02 | 1.93 |
| *Fcgr4* | 10351509 | NM_144559 | 1.39E-02 | 1.93 |
| *Ctsl* | 10410124 | NM_009984 | 1.28E-02 | 1.93 |
| *Tm6sf1* | 10554574 | NM_145375 | 1.39E-02 | 1.94 |
| *Btk* | 10606694 | NM_013482 | 1.31E-02 | 1.94 |
| *Zc3h12d* | 10361698 | NM_172785 | 3.63E-02 | 1.94 |
| *Csf1* | 10501164 | NM_007778 | 2.34E-02 | 1.94 |
| *C3ar1* | 10547657 | NM_009779 | 1.83E-02 | 1.94 |
| *---* | 10340472 | --- | 3.63E-02 | 1.95 |
| *Cd53* | 10501063 | NM_007651 | 2.14E-02 | 1.95 |
| *Nfam1* | 10430931 | NM_028728 | 1.86E-02 | 1.96 |
| *Tlr8* | 10607868 | NM_133212 | 1.53E-02 | 1.96 |
| *Myof* | 10467258 | NM_001099634 | 2.74E-02 | 1.96 |
| *Prtn3* | 10364529 | NM_011178 | 1.47E-02 | 1.96 |
| *Cotl1* | 10582162 | NM_028071 | 1.47E-02 | 1.96 |
| *Ptgr1* | 10513320 | NM_025968 | 3.09E-02 | 1.96 |
| *Alox5* | 10547153 | NM_009662 | 1.49E-02 | 1.96 |
| *Spc25* | 10483401 | NM_025565 | 1.88E-02 | 1.97 |
| *C5ar1* | 10560242 | NM_001173550 | 1.28E-02 | 1.97 |
| *Il1rap* | 10434845 | NM_008364 | 4.58E-02 | 1.97 |
| *Bace2* | 10437210 | NM_019517 | 1.06E-02 | 1.97 |
| *Cnn2* | 10364593 | NM_007725 | 7.05E-03 | 1.97 |
| *Vcan* | 10410931 | NM_001081249 | 2.94E-02 | 1.97 |
| *Dok3* | 10409502 | NM_013739 | 1.83E-02 | 1.98 |
| *F630043A04Rik* | 10420426 | NM_198605 | 4.85E-02 | 1.98 |
| *Capg* | 10539135 | NM_007599 | 1.47E-02 | 1.98 |
| *Lcp1* | 10416437 | NM_008879 | 1.28E-02 | 1.98 |
| *Nckap1l* | 10427336 | NM_153505 | 1.47E-02 | 1.99 |
| *Scn5a* | 10597656 | NM_021544 | 1.77E-02 | 1.99 |
| *Myo1f* | 10443980 | NM_053214 | 1.23E-02 | 1.99 |
| *Cd180* | 10406928 | NM_008533 | 2.96E-02 | 1.99 |
| *Fcrls* | 10499189 | NM_030707 | 1.39E-02 | 1.99 |
| *Slc38a1* | 10431874 | NM_001166456 | 2.48E-02 | 1.99 |
| *Kif18b* | 10391811 | NM_197959 | 4.89E-02 | 1.99 |
| *Mrc1* | 10469358 | NM_008625 | 2.19E-02 | 2.00 |
| *Pira1* | 10559467 | NM_011087 | 3.09E-02 | 2.00 |
| *Clec4a2* | 10541587 | NM_001170333 | 1.28E-02 | 2.00 |
| *Myo1g* | 10384154 | NM_178440 | 1.23E-02 | 2.00 |
| *Fxyd5* | 10562192 | NM_008761 | 1.28E-02 | 2.01 |
| *Nek2* | 10352767 | NM_010892 | 2.82E-02 | 2.01 |
| *Cdca5* | 10460738 | NM_026410 | 1.41E-02 | 2.01 |
| *Pdpn* | 10518147 | NM_010329 | 3.46E-02 | 2.02 |
| *Ctss* | 10494271 | NM_021281 | 1.77E-02 | 2.02 |
| *Basp1* | 10427895 | NM_027395 | 2.47E-02 | 2.03 |
| *C1qtnf6* | 10430358 | NM_028331 | 1.86E-02 | 2.04 |
| *Rnf128* | 10602009 | NM_023270 | 4.24E-02 | 2.05 |
| *Rbpj* | 10521911 | NM_001080928 | 1.23E-02 | 2.05 |
| *Npl* | 10358879 | NM_028749 | 1.84E-02 | 2.05 |
| *Zbp1* | 10490150 | NM_021394 | 3.98E-02 | 2.06 |
| *Ddah1* | 10496727 | NM_026993 | 4.82E-02 | 2.06 |
| *Sdc1* | 10394471 | NM_011519 | 1.58E-02 | 2.06 |
| *Lrrc25* | 10572445 | NM_153074 | 1.22E-02 | 2.07 |
| *Ndc80* | 10452709 | NM_023294 | 2.95E-02 | 2.07 |
| *Klk10* | 10552488 | NM_133712 | 1.39E-02 | 2.07 |
| *Emp3* | 10563441 | NM_010129 | 1.39E-02 | 2.07 |
| *B4galt5* | 10489891 | NM_019835 | 1.77E-02 | 2.07 |
| *Serpinb1a* | 10408557 | NM_025429 | 4.75E-02 | 2.07 |
| *Mcoln2* | 10496771 | NM_026656 | 2.34E-02 | 2.07 |
| *Slc16a6* | 10392440 | NM_001029842 | 1.23E-02 | 2.07 |
| *Clec12a* | 10542164 | NM_177686 | 1.47E-02 | 2.07 |
| *Gm7455* | 10596409 | NM_001167923 | 4.75E-02 | 2.09 |
| *2810417H13Rik* | 10350838 | NM_026515 | 3.17E-02 | 2.09 |
| *Nfe2l3* | 10538275 | NM_010903 | 1.83E-02 | 2.09 |
| *Sectm1a* | 10394068 | NM_145373 | 2.03E-02 | 2.09 |
| *F13a1* | 10408693 | NM_028784 | 4.50E-02 | 2.09 |
| *Slc7a8* | 10419854 | NM_016972 | 2.96E-02 | 2.10 |
| *---* | 10343794 | --- | 1.21E-02 | 2.12 |
| *Coro1a* | 10568024 | NM_009898 | 1.39E-02 | 2.12 |
| *Fam20c* | 10526853 | NM_030565 | 1.77E-02 | 2.13 |
| *Tpx2* | 10477187 | NM_001141977 | 3.71E-02 | 2.13 |
| *Abpb* | 10562314 | NM_001100464 | 2.59E-02 | 2.13 |
| *Sirpb1a* | 10497349 | NM_001002898 | 2.35E-02 | 2.13 |
| *Cd300ld* | 10392808 | NM_145437 | 1.31E-02 | 2.14 |
| *---* | 10342325 | --- | 1.23E-02 | 2.14 |
| *Sfpi1* | 10473809 | NM_011355 | 1.88E-02 | 2.14 |
| *Cd52* | 10517165 | NM_013706 | 2.46E-02 | 2.15 |
| *Tlr13* | 10601385 | NM_205820 | 2.02E-02 | 2.15 |
| *Gpr176* | 10486102 | NM_201367 | 2.85E-02 | 2.15 |
| *Gpc1* | 10348653 | NM_016696 | 2.05E-02 | 2.15 |
| *Fcgr2b* | 10360028 | NM_001077189 | 2.94E-02 | 2.15 |
| *Slc15a3* | 10461558 | NM_023044 | 1.39E-02 | 2.16 |
| *Slc39a4* | 10430006 | NM_028064 | 1.28E-02 | 2.16 |
| *Lag3* | 10547906 | NM_008479 | 1.83E-02 | 2.16 |
| *Slit2* | 10521759 | NM_178804 | 4.22E-02 | 2.17 |
| *Atp6v1c2* | 10399559 | NM_001159632 | 1.28E-02 | 2.19 |
| *Gla* | 10606714 | NM_013463 | 2.47E-02 | 2.19 |
| *Sirpb1b* | 10497364 | NM_001173460 | 1.58E-02 | 2.20 |
| *Ms4a4a* | 10461587 | XM_986941 | 3.31E-02 | 2.20 |
| *Myo18b* | 10532574 | NM_028901 | 2.78E-02 | 2.20 |
| *---* | 10340500 | --- | 4.81E-02 | 2.22 |
| *---* | 10608709 | NM_025998.1 | 4.13E-02 | 2.22 |
| *Lgals3* | 10414360 | NM_001145953 | 2.46E-02 | 2.22 |
| *Thy1* | 10584628 | NM_009382 | 1.47E-02 | 2.23 |
| *Tlr1* | 10530145 | NM_030682 | 1.87E-02 | 2.24 |
| *Lilrb3* | 10559446 | NM_011095 | 1.47E-02 | 2.25 |
| *Anln* | 10591781 | NM_028390 | 3.68E-02 | 2.25 |
| *Ncf4* | 10425053 | NM_008677 | 7.05E-03 | 2.26 |
| *Ass1* | 10363541 | NM_007494 | 1.28E-02 | 2.26 |
| *Ass1* | 10471154 | NM_007494 | 1.28E-02 | 2.26 |
| *Cyba* | 10582303 | NM_007806 | 1.06E-02 | 2.27 |
| *Stap1* | 10522788 | NM_019992 | 2.72E-02 | 2.28 |
| *Nkd2* | 10410547 | NM_028186 | 3.92E-02 | 2.28 |
| *Cthrc1* | 10423836 | NM_026778 | 3.22E-02 | 2.29 |
| *Ms4a6d* | 10466210 | NM_026835 | 1.58E-02 | 2.29 |
| *Ms4a6b* | 10461622 | NM_027209 | 1.47E-02 | 2.30 |
| *E2f8* | 10563780 | NM_001013368 | 3.29E-02 | 2.31 |
| *Fgr* | 10508772 | NM_010208 | 1.83E-02 | 2.32 |
| *Plaur* | 10550906 | NM_011113 | 2.85E-02 | 2.33 |
| *Ccr2* | 10590631 | NM_009915 | 1.30E-02 | 2.33 |
| *AU018091* | 10569687 | NM_001004153 | 1.88E-02 | 2.33 |
| *Pilra* | 10534927 | NM_153510 | 2.44E-02 | 2.34 |
| *Mastl* | 10480432 | NM_025979 | 2.93E-02 | 2.34 |
| *Alox5ap* | 10527638 | NM_009663 | 1.28E-02 | 2.34 |
| *Kif23* | 10594251 | NM_024245 | 2.90E-02 | 2.34 |
| *Il4ra* | 10557326 | NM_001008700 | 2.75E-02 | 2.34 |
| *Fxyd6* | 10584883 | NM_022004 | 1.28E-02 | 2.34 |
| *Scube1* | 10431051 | NM_022723 | 2.17E-02 | 2.36 |
| *Casc5* | 10474875 | NM_029617 | 3.98E-02 | 2.37 |
| *Adamts4* | 10351551 | NM_172845 | 4.05E-02 | 2.38 |
| *Myo18b* | 10532586 | NM_028901 | 2.72E-02 | 2.38 |
| *Fpr1* | 10448124 | NM_013521 | 4.45E-02 | 2.40 |
| *Tmem8* | 10442932 | NM_021793 | 2.78E-02 | 2.40 |
| *---* | 10344285 | --- | 4.90E-02 | 2.41 |
| *Cyp7b1* | 10497381 | NM_007825 | 2.95E-02 | 2.42 |
| *Ms4a6c* | 10461614 | NM_028595 | 1.26E-02 | 2.43 |
| *Aurkb* | 10377405 | NM_011496 | 2.15E-02 | 2.44 |
| *---* | 10339054 | --- | 4.24E-02 | 2.44 |
| *Emb* | 10407327 | NM_010330 | 1.23E-02 | 2.45 |
| *Kif2c* | 10515431 | NM_134471 | 2.38E-02 | 2.45 |
| *Gpr183* | 10422496 | NM_183031 | 1.39E-02 | 2.45 |
| *Ccl6* | 10389222 | NM_009139 | 2.62E-02 | 2.46 |
| *Tnfsf8* | 10513729 | NM_009403 | 2.73E-02 | 2.47 |
| *Myo18b* | 10532578 | NM_028901 | 1.41E-02 | 2.47 |
| *Fam3c* | 10543319 | NM_138587 | 4.01E-04 | 2.48 |
| *Lrg1* | 10451953 | NM_029796 | 2.23E-02 | 2.48 |
| *Ccr5* | 10590635 | NM_009917 | 3.56E-03 | 2.49 |
| *Ccr5* | 10598013 | NM_009917 | 3.56E-03 | 2.49 |
| *Sirpb1b* | 10497358 | NM_001173460 | 1.97E-02 | 2.49 |
| *Fcgr1* | 10500335 | NM_010186 | 1.28E-02 | 2.50 |
| *F10* | 10570291 | NM_007972 | 2.75E-02 | 2.52 |
| *Cd72* | 10512470 | NM_001110320 | 2.34E-02 | 2.52 |
| *Atp6v0d2* | 10511779 | NM_175406 | 1.39E-02 | 2.54 |
| *Rbp1* | 10588037 | NM_011254 | 3.09E-02 | 2.56 |
| *Fabp7* | 10363224 | NM_021272 | 4.79E-02 | 2.57 |
| *Itgb2* | 10364262 | NM_008404 | 8.66E-03 | 2.59 |
| *Il7r* | 10427628 | NM_008372 | 2.77E-02 | 2.59 |
| *Gm885* | 10382106 | NM_001033435 | 3.56E-03 | 2.63 |
| *Hist1h2ab* | 10404063 | NM_175660 | 3.40E-02 | 2.64 |
| *Cep55* | 10462866 | NM_001164362 | 3.29E-02 | 2.64 |
| *Mmp3* | 10583071 | NM_010809 | 2.39E-02 | 2.64 |
| *2810417H13Rik* | 10586448 | NM_026515 | 2.96E-02 | 2.65 |
| *Adam8* | 10568873 | NM_007403 | 2.46E-02 | 2.65 |
| *Itgax* | 10557895 | NM_021334 | 1.58E-02 | 2.67 |
| *C6* | 10422635 | NM_016704 | 2.33E-02 | 2.68 |
| *S100a4* | 10493812 | NM_011311 | 1.98E-02 | 2.69 |
| *Sectm1b* | 10394060 | NM_026907 | 3.21E-02 | 2.69 |
| *Gpnmb* | 10538187 | NM_053110 | 1.23E-02 | 2.72 |
| *Ccl9* | 10389214 | NM_011338 | 1.28E-02 | 2.73 |
| *Epb4.1l4a* | 10458052 | NM_013512 | 1.28E-02 | 2.74 |
| *Plek* | 10384458 | NM_019549 | 1.47E-02 | 2.74 |
| *Slc7a11* | 10498024 | NM_011990 | 3.80E-02 | 2.75 |
| *Bub1* | 10487480 | NM_001113179 | 2.93E-02 | 2.77 |
| *Egr2* | 10363735 | NM_010118 | 1.58E-02 | 2.78 |
| *Clec4n* | 10541605 | NM_020001 | 3.22E-02 | 2.81 |
| *Gjb2* | 10420362 | NM_008125 | 1.23E-02 | 2.83 |
| *Has2* | 10428707 | NM_008216 | 4.05E-02 | 2.83 |
| *Mmp12* | 10583056 | NM_008605 | 1.28E-02 | 2.84 |
| *Ccl7* | 10379518 | NM_013654 | 1.47E-02 | 2.84 |
| *Gm5150* | 10497372 | DQ055451 | 1.26E-02 | 2.85 |
| *Hpgd* | 10571840 | NM_008278 | 2.02E-02 | 2.88 |
| *Slc37a2* | 10592266 | NM_001145960 | 1.23E-02 | 2.92 |
| *Bst1* | 10521667 | NM_009763 | 2.02E-02 | 2.97 |
| *Ccl12* | 10379530 | NM_011331 | 1.06E-02 | 2.97 |
| *Dpep2* | 10581434 | NM_176913 | 8.66E-03 | 3.03 |
| *Vsig4* | 10605848 | NM_177789 | 4.75E-02 | 3.10 |
| *F7* | 10570280 | NM_010172 | 1.28E-02 | 3.11 |
| *Spon1* | 10556509 | NM_145584 | 3.35E-02 | 3.11 |
| *Clec4d* | 10541614 | NM_010819 | 3.82E-02 | 3.11 |
| *Cldn4* | 10534395 | NM_009903 | 1.88E-02 | 3.18 |
| *Cpxm1* | 10487645 | NM_019696 | 1.21E-02 | 3.21 |
| *Serpine2* | 10355984 | NM_009255 | 1.28E-02 | 3.27 |
| *Ccr1* | 10598004 | NM_009912 | 1.12E-02 | 3.30 |
| *Msr1* | 10578264 | NM_031195 | 1.28E-02 | 3.43 |
| *Itgam* | 10557862 | NM_001082960 | 7.05E-03 | 3.50 |
| *BC055004* | 10526783 | NM_001013773 | 1.38E-02 | 3.50 |
| *Il1f9* | 10469786 | NM_153511 | 4.82E-02 | 3.69 |
| *4930578G10Rik* | 10504203 | ENSMUST00000107984 | 1.88E-02 | 3.73 |
| *Mmp13* | 10583044 | NM_008607 | 1.21E-02 | 3.93 |
| *Ms4a4c* | 10461594 | NM_029499 | 2.14E-02 | 3.95 |
| *Reg1* | 10539200 | NM_009042 | 4.99E-02 | 4.04 |
| *Ccl8* | 10379535 | NM_021443 | 2.78E-02 | 4.05 |
| *Clec5a* | 10544273 | NM_001038604 | 7.05E-03 | 4.10 |
| *Saa1* | 10563611 | NM_009117 | 3.09E-02 | 4.11 |
| *Muc4* | 10435112 | NM_080457 | 2.16E-03 | 4.19 |
| *Mmp8* | 10583100 | NM_008611 | 3.01E-02 | 4.25 |
| *Il1rn* | 10469816 | NM_031167 | 2.34E-02 | 4.76 |
| *Tm4sf19* | 10435043 | NM_001160402 | 1.21E-02 | 4.82 |
| *Saa3* | 10563597 | NM_011315 | 4.24E-02 | 5.44 |
| *Timp1* | 10598976 | NM_001044384 | 2.02E-02 | 5.51 |
| *Clec4e* | 10547664 | NM_019948 | 1.47E-02 | 6.61 |
| *Crabp1* | 10585438 | NM_013496 | 4.22E-02 | 6.70 |
| *Gm10573* | 10516042 | ENSMUST00000097904 | 1.23E-02 | 7.25 |
| *Mir30b* | 10429197 | NR_029534 | 3.56E-03 | 12.12 |
